# Analysis of triglyceride synthesis unveils a green algal soluble diacylglycerol acyltransferase and provides clues to potential enzymatic components of the chloroplast pathway

**DOI:** 10.1186/s12864-017-3602-0

**Published:** 2017-03-09

**Authors:** Carolina Bagnato, María B. Prados, Gisela R. Franchini, Natalia Scaglia, Silvia E. Miranda, María V. Beligni

**Affiliations:** 1 Instituto de Energía y Desarrollo Sustentable, Comisión Nacional de Energía Atómica, Centro Atómico Bariloche, Av. Bustillo 9500, 8400S. C. de Bariloche, Río Negro, Argentina; 2 Instituto de Investigaciones Bioquímicas de La Plata (INIBIOLP-CONICET-UNLP), Facultad de Ciencias Médicas, Universidad Nacional de La Plata, Calle 60 y 120 s/n, 1900 La Plata, Argentina; 3 Universidad de Buenos Aires. CONICET Instituto de Investigaciones Cardiológicas (ININCA), − Laboratorio de Glico-Inmuno-Biología, Marcelo T. de Alvear 2270, C1122AAJ, Buenos Aires, Argentina; 4 Instituto de Investigaciones Biológicas (IIB-CONICET-UNMdP), Facultad de Ciencias Exactas y Naturales, Universidad Nacional de Mar del Plata, CC 1245, 7600 Mar del Plata, Argentina

**Keywords:** Algae, Biodiesel production, Chloroplast, HMMER profiling, Neutral lipids, Soluble acyltransferase, Protein phylogeny, Triglyceride metabolism

## Abstract

**Background:**

Microalgal triglyceride (TAG) synthesis has attracted considerable attention. Particular emphasis has been put towards characterizing the algal homologs of the canonical rate-limiting enzymes, diacylglycerol acyltransferase (DGAT) and phospholipid:diacylglycerol acyltransferase (PDAT). Less work has been done to analyze homologs from a phylogenetic perspective. In this work, we used HMMER iterative profiling and phylogenetic and functional analyses to determine the number and sequence characteristics of algal DGAT and PDAT, as well as related sequences that constitute their corresponding superfamilies. We included most algae with available genomes, as well as representative eukaryotic and prokaryotic species.

**Results:**

Amongst our main findings, we identified a novel clade of DGAT1-like proteins exclusive to red algae and glaucophyta and a previously uncharacterized subclade of DGAT2 proteins with an unusual number of transmembrane segments. Our analysis also revealed the existence of a novel DGAT exclusive to green algae with moderate similarity to plant soluble DGAT3. The DGAT3 clade shares a most recent ancestor with a group of uncharacterized proteins from cyanobacteria. Subcellular targeting prediction suggests that most green algal DGAT3 proteins are imported to the chloroplast, evidencing that the green algal chloroplast might have a soluble pathway for the *de novo* synthesis of TAGs. Heterologous expression of *C. reinhardtii* DGAT3 produces an increase in the accumulation of TAG, as evidenced by thin layer chromatography.

**Conclusions:**

Our analysis contributes to advance in the knowledge of complex superfamilies involved in lipid metabolism and provides clues to possible enzymatic players of chloroplast TAG synthesis.

**Electronic supplementary material:**

The online version of this article (doi:10.1186/s12864-017-3602-0) contains supplementary material, which is available to authorized users.

## Background

There is currently great recognition of the potential of microalgae for the production of biodiesel. However, the difficulties in maintaining elevated culture biomass and oil productivity create bottlenecks that hamper the economic viability of the process [1–3]. Considerable work has been done to improve the conditions of microalgal culture in order to increase oil production [4–8]. A concluding remark of many experiments is that most species increase their triglyceride (TAG) content under stress, such us nitrogen deprivation [9–12]. The challenge is that TAG accumulation seems to occur in conjunction with a reduction in cell proliferation, with a consequent decrease in total oil productivity. In this scenario, genetic engineering of lipid metabolism could constitute a powerful tool to overcome these limitations. However, following this route is not trivial, since a profound understanding of lipid metabolic pathways is required in order to tailor genetic engineering tools. This is particularly true for algae, organisms for which our knowledge about TAG metabolism is incomplete and mostly inferred. First, algae are a paraphyletic group with representatives of several different kingdoms or supergroups [13]. Second, not all algae accumulate TAGs as their main storage compound [14–16], hence it is expected that this creates fundamental differences in the regulation of the synthesizing pathways. Third, even for the more-closely related green algae, recent studies have put in evidence interesting differences between microalgal and plant TAG metabolism [17, 18].

TAGs are the major molecules of energy storage in most eukaryotes. There are several accepted pathways for the synthesis of TAGs. One of the best characterized is the Kennedy pathway, which implies the sequential acylation of fatty acids (usually activated in the form of acyl-CoA) on a glycerol-3-phosphate backbone. In animals, yeasts and plants, in which it has been best characterized, this pathway is located in the endoplasmic reticulum, evidenced by subcellular localization studies of the main participating enzymes [19–21]. The final step, the acylation of acyl-CoA onto a molecule of sn-1,2-diacylglycerol (DAG) is the rate-limiting step, and is catalyzed by several types of diacylglycerol acyltransferases (DGATs). Most DGATs are integral microsomal membrane proteins. The most numerous and best characterized forms in mammals [22, 23] and plants [21] are DGAT1 and DGAT2, which, despite sharing a few amino acids, show overall little sequence similarity with one another. In addition, other DGAT forms were reported in particular tissues of certain plants [24–26].

In yeasts and plants, TAG biosynthesis can also occur via phospholipid (PL) remodeling, via the action of CoA-independent transacylases [27]. The best characterized is membrane-bound phospholipid: diacylglycerol acyltransferase (PDAT). PDAT catalyzes the transfer of a fatty acyl moiety from the sn-2-position of a PL to the sn-3-position of sn-1, 2- DAG, thus forming TAG and a lyso-phospholipid [27, 28].

Several structural and evolutionary studies have been done for DGATs [29–32] and PDATs [27, 33], but only a scarce amount have tried to analyze algal homologs from different supergroups in a phylogenetic context [34, 35]. One of the key steps towards characterizing microalgal TAG synthesis pathway refers to the proper annotation of enzymes. Most enzymes are initially identified through in silico similarity searches using canonical enzymes with biochemical evidence as templates. Most of these enzymes are from plants, yeasts and animals, which not always represent the best dataset to search for homologs in unrelated organisms, such as heterokonts. In addition, the tools most commonly used, such as BLAST, use limited information to perform a search and are not always effective at identifying distant homologs. Other tools, such as HMMER, PHI-BLAST or a combination of methods in an iterative form, include more parameters into the search and are, hence, more sensitive than BLAST [36–38].

With the purpose of identifying all possible enzymes within the TAG synthesis pathway in algae and analyzing them in a phylogenetic context, we performed an in-depth sequence data mining. We followed a HMMER-iterative strategy that included 26 algae and 53 related and non-related eukaryotic species, as well as representative prokaryotic species. Phylogeny and standard biocomputational analyses were performed in order to make testable predictions of the characteristics of the encoded proteins in different algae taxa. Our results show some interesting new findings about several of the enzymes analyzed. One of the most significant refers to the identification of a novel DGAT exclusive to green algae that shows moderate similarity to DGAT3, a soluble DGAT previously characterized in plants [25, 39, 40]. Subcellular localization prediction suggests that most green algal DGAT3 proteins likely localize to the chloroplast. Phylogenetic analysis evidences that the DGAT3 clade shares a most recent ancestor with a group of uncharacterized proteins from cyanobacteria. Heterologous expression of *C. reinhardtii* DGAT3 produced an increase in the accumulation of TAGs in *E. coli* cells compared to control bacteria. Altogether, our analysis provides useful information about the characteristics and phylogenetic relationships of the main enzymes that participate in TAG synthesis. The implications of the existence of a green algal DGAT3, as well as its possible involvement in chloroplast TAG synthesis, is discussed.

## Results

With the purpose of annotating the TAG synthesis pathway in algae following a phylogenetic perspective and trying to identify novel homologs, we did a HMMER iterative data mining. We used the predicted proteomes from most algae with available sequenced genomes (Table 1), as well as from a set of representative eukaryotic organisms (Additional file 1). In order to do the analyses in a true phylogenetic context, we also searched for prokaryotic homologs in the HMMER website. Although this procedure was done for PDAT and for all the enzymes of the *de novo* synthesis of TAGs, we mainly focused on the enzymes of the rate-limiting steps, PDAT and DGATs.

**Table 1:** Algae species used for computational analysis of the TAG pathway

Classification at Higher Ranks^a^		Lower Ranks	Code^b^
ARCHAEPLASTIDA	Glaucophyta	*Cyanophora paradoxa*	Cypa
	Rhodophyta (Red Algae)	*Cyanidioschyzon merolae*	Cmer
		*Galderia sulphuraria*	Gsul
		*Chondrus crispus*	Ccri
		*Porphyridium purpureum*	Ppur
	Chloroplastida	Chlorophyta (Green Algae)	
		*Coccomyxa subellipsoidea*	Csub
		*Chlorella variabilis*	Cvar
		*Chlamydomonas reinhardtii*	Crei
		*Volvox carteri*	Vcar
		*Bathycoccus prasinos*	Bpra
		*Micromonas pusilla*	Mpus
		*Micromonas sp.*	Misp
		*Ostreococcus lucimarinus*	Oluc
		*Ostreococcus tauri*	Otau
		Streptophyta (Plants)	
SAR	Rhizaria	*Bigelowiella natans*	Bnat
	Alveolata	Dinoflagellata (Dinoflagellates)	
		*Symbiodinium minutum*	Smin
	Stramenopiles	Eustigmatales	
		*Nannochloropsis gaditana*	Ngad
		*Nannochloropsis oceanica*	Noce
		Pelagophyceae	
		*Aureococcus anophagefferens*	Aano
		Phaeophyceae (Brown Algae)	
		*Ectocarpus siliculosus*	Esil
		Diatomea (Diatoms)	
		*Fragilariopsis cylindrus*	Fcyl
		*Pseudo-nitzschia multiseries*	Pmul
		*Phaeodactylum tricornutum*	Ptri
		*Thalassiosira pseudonana*	Tpse
Haptophyta		*Emiliania huxleyi*	Ehux
Cryptophyceae		*Guillardia theta*	Gthe

^a^ Classification at higher ranks is according to Adl et al. (2012)

^b^ Internal species codes used for computational analysis

### PDAT in-silico analyses

PDATs (EC:2.3.1.158) belong to a superfamily that is also composed of lecithin:cholesterol acyltransferases (LCATs) (EC: 2.3.1.43), enzymes that catalyze the transacylation of acyl groups from phospholipids to a variety of sterols [33]. *Arabidopsis thaliana* has two reported PDATs (PDAT1 and PDAT2) [28] and four LCAT-like proteins. LCAT2 is a phospholipid:sterol acyltransferase (PSAT) involved in sterol ester synthesis [41], whereas LCAT1, LCAT3 and LCAT4 mainly participate in phospholipid catabolism [42].

**Fig. 1:**
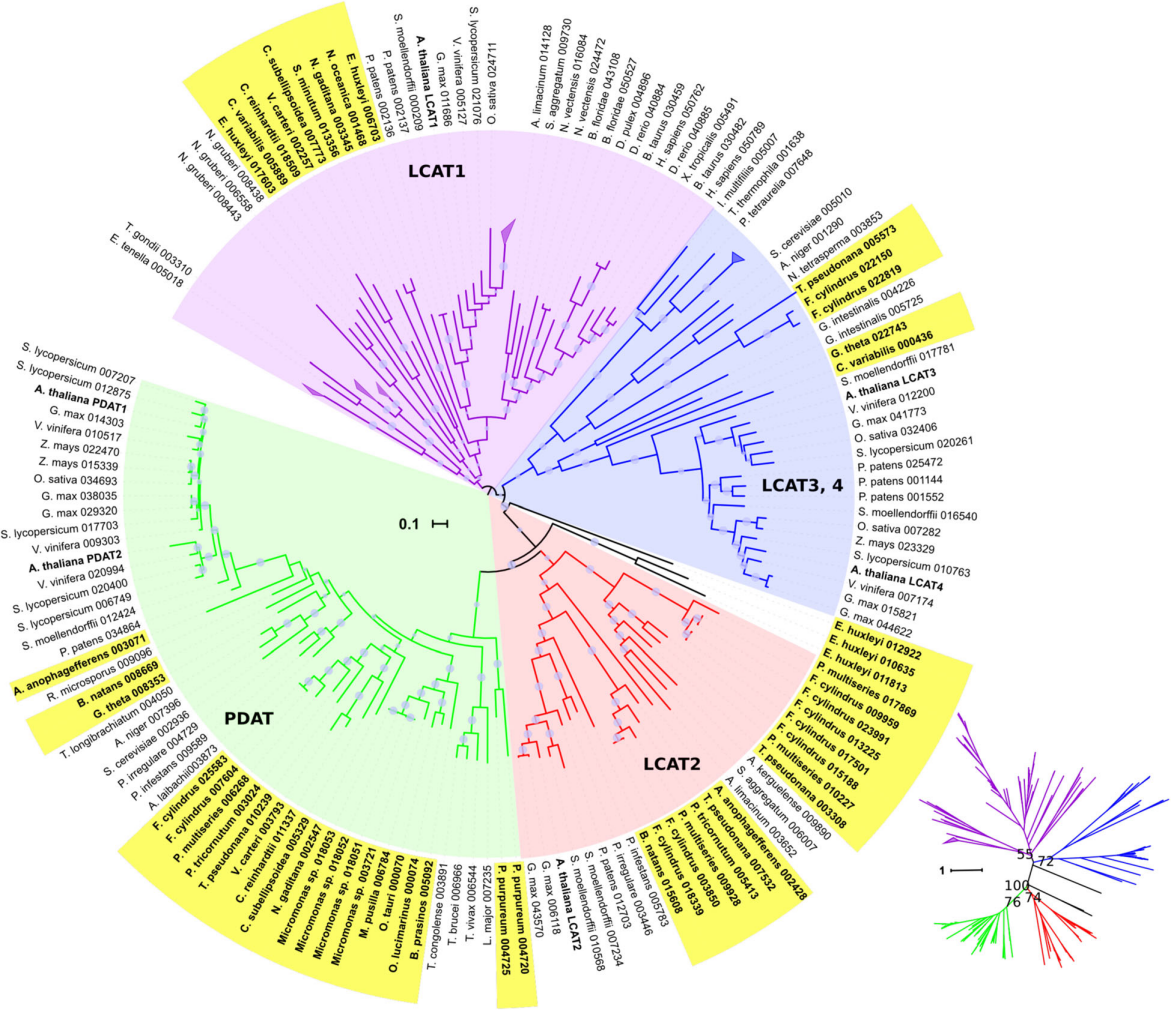
Phylogenetic relationships of algae members of the LCAT superfamily. Rooted circular phylogram representation of the consensus tree generated by the maximum likelihood (ML) method (500 bootstraps) on the conserved regions of proteins from the LCAT superfamily of the species detailed in Table 1 and Additional file 1. *Gray circles* represent ML bootstrap values > 50. Labels highlighted in *yellow* are algal sequences, numbers after the abbreviated species names are internal IDs. *A. thalian*a proteins are shown in bold for reference. The scale bar represents 0.1 amino acid substitution per site. A few clades were collapsed for simplicity. The inset is the same tree as an unrooted radial phylogram, with the ML bootstrap values for the four main clades: PDAT, LCAT1, LCAT2 and LCAT3-4. For the inset, the scale bar represents 1 amino acid substitution per site. To view the trimmed MSA used to reconstruct the tree in Phylip format and the raw consensus tree in Newick format, see Additional file 6

For a proper characterization of the TAG pathway, it is fundamental to distinguish between PDAT and LCAT activities, since most LCATs biochemically characterized are not active with neutral lipids. With that purpose, we generated an LCAT *Hidden Markov Model* (called profile hmm) that would allow for the identification of all the members of the superfamily, while using subsequent phylogenetic and clustering analyses to classify the superfamily members to each one of the groups. Figure 1 shows a phylogentetic tree of PDATs and LCATs from algae and other representative taxa. Table 2 summarizes the number of PDATs identified in algae, while Additional file 2 shows a detail of all the LCAT superfamily homologs identified. Among green algae, Chlorophyceae have one PDAT and one LCAT1 homolog, whereas Mamiellophyceae do not appear to have LCATs. LCAT2 homologs seem to be unique to SAR algae. In fact, diatoms show LCAT2 and no LCAT1 homologs. PDAT and LCAT2 appear to have evolved from a most recent common ancestor, evidenced by the statistical support of the bifurcation (ML bootstrap value = 100).

**Table 2:** Number and predicted chloroplast localization^a^ of DGAT1, DGAT2, DGAT3 and PDAT sequences identified in algae^b^ by HMMER iterative data mining

Algae				DGAT1	DGAT2^e^	DGAT3	PDAT1, 2
Archaeplastida	Glaucophyta		*Cyanophora paradoxa*	1^c^	1	—	1
	Red Algae	Cyanidiophyceae	*Cyanidioschyzon merolae*	1^c^	1	—	—
			*Galdiera sulphuraria*	1^c^	1	—	—
		Florideophyceae	*Chondrus crispus*	3^c,d^	—	—	—
		Porphyridiophyceae	*Porphyridium purpureum*	1^c^	1	—	2
	Green Algae	Trebouxiophyceae	*Coccomyxa subellipsoidea*	1	4	1	1
			*Chlorella variabilis*	1	4	***1-CH***	--
		Chlorophyceae	*Chlamydomonas reinhardtii*	***1-CH***	6	***1-CH***	***1-CH***
			*Volvox carteri f. nagariensis*	1	4	***1-CH***	1
		Mamiellophyceae	*Bathycoccus prasinos*	—	***3-CH***	***1-CH***	***1-CH***
			*Micromonas pusilla*	—	***4-CH***	***1-CH***	***1-CH***
			*Micromonas sp. RCC299*	—	6	***1-CH***	4^d^
			*Ostreococcus lucimarinus*	—	4	***1-CH***	1
			*Ostreococcus tauri*	—	***4-CH***	1	1
SAR	Rhizaria		*Bigelowiella natans*	—	6	—	1
	Alveolata	Dinoflagellata	*Symbiodinium minutum*	—	17	—	1
	Stramenopiles	Eustigmatales	*Nannochloropsis gaditana*	1	8	—	1
			*Nannochloropsis oceanica*	1	11	—	1
		Pelagophyceae	*Aureococcus anophagefferens*	1	5	—	2
		Phaeophyceae	*Ectocarpus siliculosus*	1	6	—	--
		Diatoms	*Fragilariopsis cylindrus*	2^d^	4	—	2
			*Pseudo-nitzschia multiseries*	—	4	—	1
			*Phaeodactylum tricornutum*	1	4	—	1
			*Thalassiosira pseudonana*	1	3	—	1
Haptophyta			*Emiliania huxleyi*	1	7	—	1
Cryptophyceae			*Guillardia theta*	1	3	—	1

^a^ Sequences with predicted chloroplast targeting are indicated with bold, italicized numbers followed by CH. ^b^ SAR, Stramenopiles-Alveolata-Rhizaria. ^c^ DGAT1-like clade. ^d^ These proteins are very similar to each other and correspond to gene models coming from the same contig region. They are most likely one protein. ^e^ For DGAT2, only full-length proteins are reported here

**Fig. 2:**
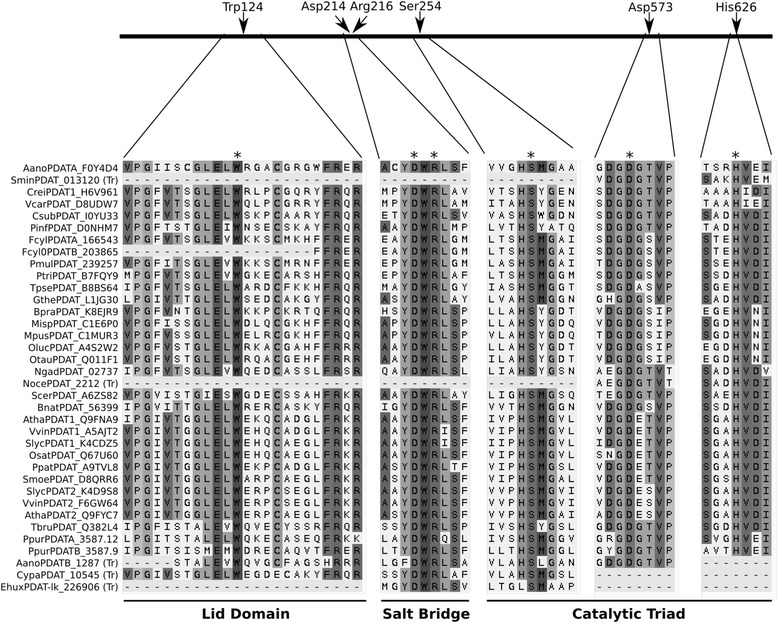
MSA of conserved regions of PDATs from algae and examples from other representative taxa. The black line represents *A. thaliana* PDAT, *arrows* and *asterisks* point to important amino acids within the structurally conserved elements: the so-called lid region, the salt bridge and the catalytic triad, based on human LCAT [43, 44] and the analysis of plant PDATs done by Pan et al. [33]. Sequence order corresponds to MAFFT alignment. Species are indicated with their internal codes, first letter of the genus and first three letters of the species (see Additional file 1 for details). IDs correspond to Uniprot IDs when available or to specific genome IDs when the sequence was not found in Uniprot. (Tr) indicates that the corresponding sequence is truncated in any of the conserved structural elements

It was reported previously that human LCAT contains several structurally conserved elements [43, 44], including a catalytic triad of Ser-181-His-377-Asp-345, a salt bridge between Asp-145 and Arg-147, and a so-called lid region. These features, as well as the conserved amino acids, were also identified in plant PDATs [33]. For data mining, we used these conserved motifs to scrutinize sequences and correct MSAs. Figure 2 shows that most of the sequences identified have all the motifs, whereas a few are truncated in either the amino or carboxy end. Since gaps in MSAs are considered ambiguity, some of these last sequences were not used for phylogenetic tree reconstruction. In these cases, clustering within the PDAT or LCAT group was determined by examining the scores obtained when the sequences were confronted with group-specific profile hmms. The MSA in Fig. 2 shows the conserved motifs that are most important for activity in all algae PDATs, as well as others from representative taxa.

### DGAT1 in-silico analyses

DGAT1 is part of a clan of proteins named MBOAT (for membrane bound O-acyl transferase) that contains a variety of membrane acyltransferase enzymes. One of the superfamilies within the MBOAT clan is the sterol O-acyltransferase (SOAT) group (Interpro family IPR014371), which in fact contains three different families. The first one is the SOAT/ACAT family, whose best characterized members are mammalian SOAT1 and SOAT2 (also known as ACAT1 and ACAT2), which play a role in the formation of fatty acid-cholesterol esters [45]. The second is the ARE family (for acyl-coenzyme A: cholesterol acyl transferase-related enzyme), which contains the fungal homologs of animal SOATs [46]. The third family is the diacylglycerol O-acyltransferase 1 (DGAT1) family.

**Fig. 3:**
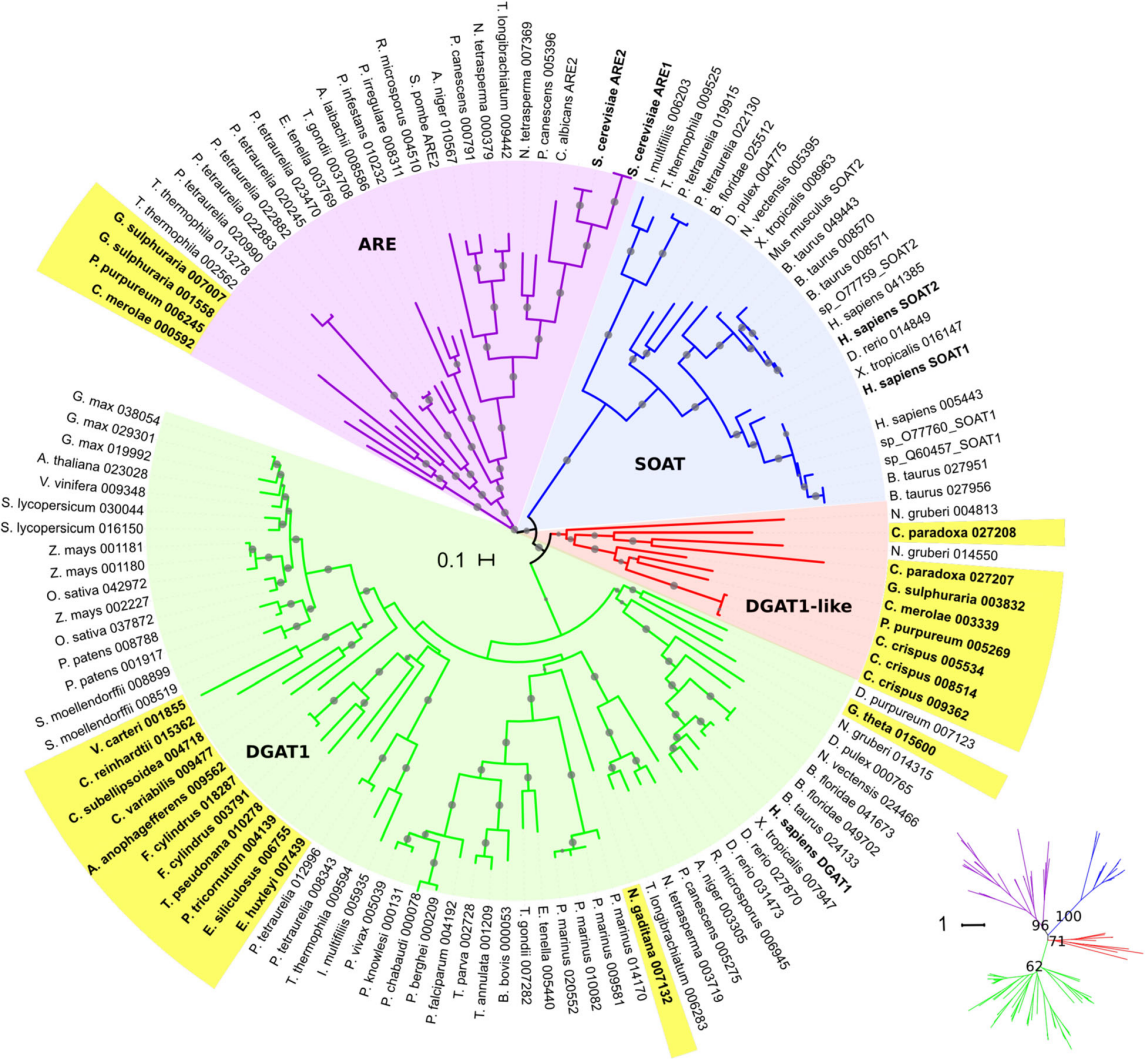
Phylogenetic relationships of algae members of the MBOAT superfamily. Rooted circular phylogram representation of the tree generated by the maximum likelihood (ML) method (500 bootstraps) on the conserved regions of proteins from the MBOAT superfamily of the species detailed in Table 1 and Additional file 1. *Gray circles* represent ML bootstrap values > 50. Labels highlighted in *yellow* are algal sequences, numbers after the abbreviated species names are internal IDs. *Homo sapiens* and *Saccharomyces cerevisiae* proteins are shown in *bold* for reference. Other SOAT and ARE proteins from species not included in Additional file 1 were also added to the analysis and are shown by their Uniprot IDs. The *scale bar* represents 0.1 amino acid substitution per site. A few clades were collapsed for simplicity. The inset is the same tree as an unrooted radial phylogram, with the ML bootstrap values for the four main clades: SOAT, ARE, DGAT1 and DGAT1-like. For the inset, the *scale bar* represents 1 amino acid substitution per site. To view the trimmed MSA used to reconstruct the tree in Phylip format and the raw consensus tree in Newick format, see Additional file 7

**Fig. 4:**
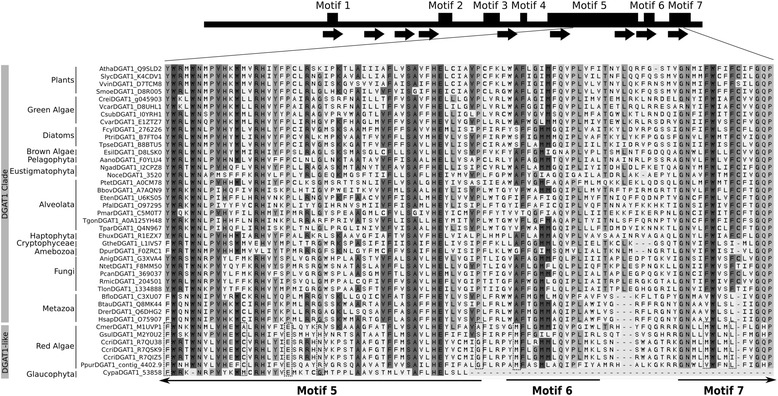
MSA of selected conserved regions of DGAT1s from algae and examples from other representative taxa. The *black horizontal arrows* represent *A. thaliana* DGAT1 and the predicted transmembrane domains, respectively. Motifs 1–7 correspond to the conserved regions defined by Cao [29]. The sequences shown cluster either into the DGAT1 clade or the DGAT1-like clade. The alignment corresponds to motif 6 and parts of motifs 5 and 7, where the main differences between the sequences within the DGAT1-like clade and the rest of the DGAT1s in the Archaeplasida supergroup lie. The level of conservation increases from *light gray* to *dark gray*. The *dashed boxes* depict the most remarkable dissimilarities between the DGAT1-like clade and their DGAT1 clade homologs from the Archaeplastida group. Sequences are indicated with their internal codes, first letter of the genus and first three letters of the species (see Additional file 1 for details). IDs correspond to Uniprot IDs when available or to specific genome IDs when the sequence was not found in Uniprot. Taxonomic classification at higher ranks is shown on the *left*

Figure 3 shows a phylogentic tree of the SOAT/ARE/DGAT superfamily. The SOAT clade contains all the SOATs from animals, and a related subclade of SOAT-like proteins from ciliate protists. The ARE clade contains the previously characterized enzymes from yeast and other fungi, as well as several proteins from non-photosynthetic protists and from red algae. Last, the DGAT1 clade contains members from most algae analyzed, as well as homologous proteins from many related and unrelated organisms within the tree of life. Amongst green algae, all chloropycean and trebouxiophycean species analyzed show a single DGAT1, whereas mamiellophycean species appear not to have DGAT1 (Table 2). From SAR algae, only *Symbiodinium minutum* (Alveolata), *Bigellowiella natans* (Rhizaria) and the diatom *Pseudo-nitzschia multiseries* appear not to have DGAT1. Interestingly, there is a fourth clade in the superfamily constituted exclusively of proteins from red algae and glaucophyta and from the heterolobosean *Naegleria gruberi* (Fig. 3)*.* When confronting the proteins within this clade to DGAT1-, ARE- and SOAT -specific profile hmms, all the members of this group show very similar scores to both ARE and DGAT1 proteins (data not shown). Considering that red algae have ARE homologs, these proteins could be DGAT1 proteins that have diverged significantly from conventional DGAT1s. Hence, we termed this the DGAT1-like Clade. Indeed, phylogenetic analysis shows that the DGAT and DGAT1-like clades are related by a most recent common ancestor (ML bootstrap support = 100), further supporting the idea that the members of the DGAT1-like clade might have DGAT1 activity. The main differences in the conserved amino acids, as described by Cao et al. [29] between this clade and the DGAT1 group lie within motifs 5 and 7 (Fig. 4). Some of the modifications seem to be unique to this clade, such as a conserved proline (P) in motif 5 of DGAT1s that is replaced with a glutamic acid (E) only in the DGAT1-like clade. Other differences are shared by other non-algal proteins, mainly metazoans, such as two phenylalanine (F) replacements to methionine (M), leucine (L) or valine (V) in motif 7.

### DGAT2 in-silico analyses

**Fig. 5:**
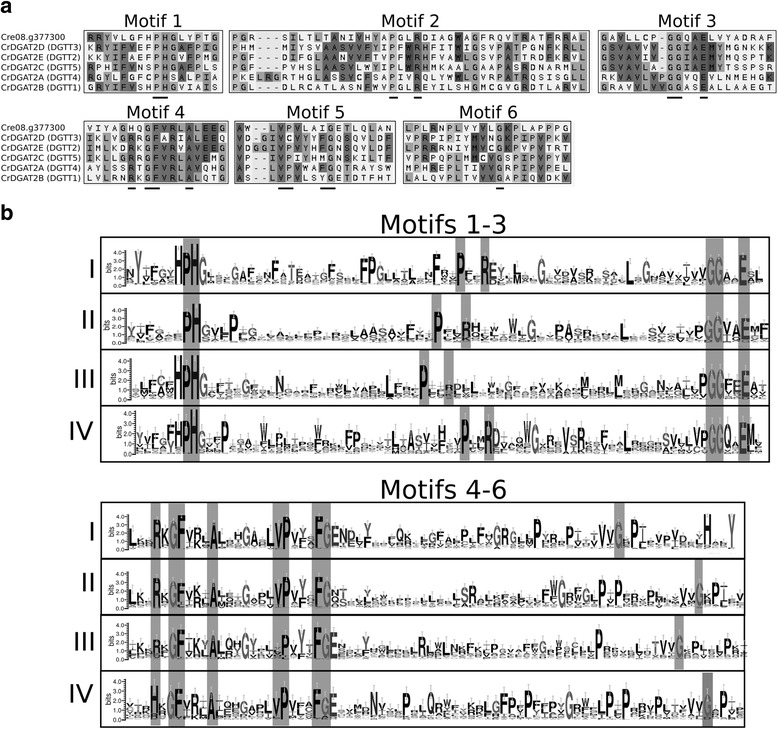
Sequence features of algal DGAT2 proteins. **a** MSA of the six *C. reinhardtii* DGAT2 identified by data mining. The six conserved motifs are shown. The level of residue conservation increases from *light gray* to *dark gray. Underlined* amino acids correspond to the completely conserved residues defined by Cao [29]. **b** Sequence logos of motifs 1–6 for the four main clades (I-IV) of the DGAT2 family. The *y* axes show the logo bits; error bars are shown on each position. Completely conserved residues are *highlighted*

DGAT2 is represented by Pfam DAGAT family. It belongs to the Pfam clan named Acyltransferases (CL0228), which also contains glycerol-3-phosphate acyltransferase (GPAT), acylglycerol-3-phosphate acyltransferase (AGPAT) and other protein families with whom DGAT2 has a modest similarity. DGAT2 is by far the most abundant DGAT in algae [34, 47]. The precise number of homologs of each species cannot be unambiguously determined, since many of the sequences identified using sensitive data-mining are almost identical to each other, complicated by the fact that many genomes do not have extensive curation and have several examples of redundant predicted proteins coming from the same gene model. Many of the sequences identified for DGAT2 contain small replacements in conserved residues that are shared among a group of proteins, raising the question of whether those replacements might be functional. Others are truncated and cannot be used for phylogenetic tree reconstruction. Since the similarity between DGAT2 and the rest of the acyltransferase clan is indeed limited, we did not analyze the phylogenetic relationships of the superfamily. In this case, we retrieved the sequences from algae that had all the conserved motifs (Table 2 and Additional file 3), as described by Cao et al. [29], even if they had small replacements. For instance, we identified six DGAT2s in *C. reinhardtii* (Fig. 5a), while historically there had been five homologs reported (DGTT1-DGTT5). Two of them have all the completely conserved residues (DGTT2 and DGTT4), while the rest show replacements at different residues of motif five (V), the so-called VPFG block. DGTT5 and Cre08.g377300 have an F to I or to M modification, DGTT3 has a P to C replacement and DGTT1 has an F to Y modification. It is noteworthy that DGTT1 (CrDGAT2B) has biochemical evidence [9, 48], which indicates that the F to Y replacement results in an active protein. Hence, so far we cannot discard sequences that show small differences in the “completely conserved” residues, since amino acid replacement may not necessarily result in a loss of activity [9, 49].

**Fig. 6:**
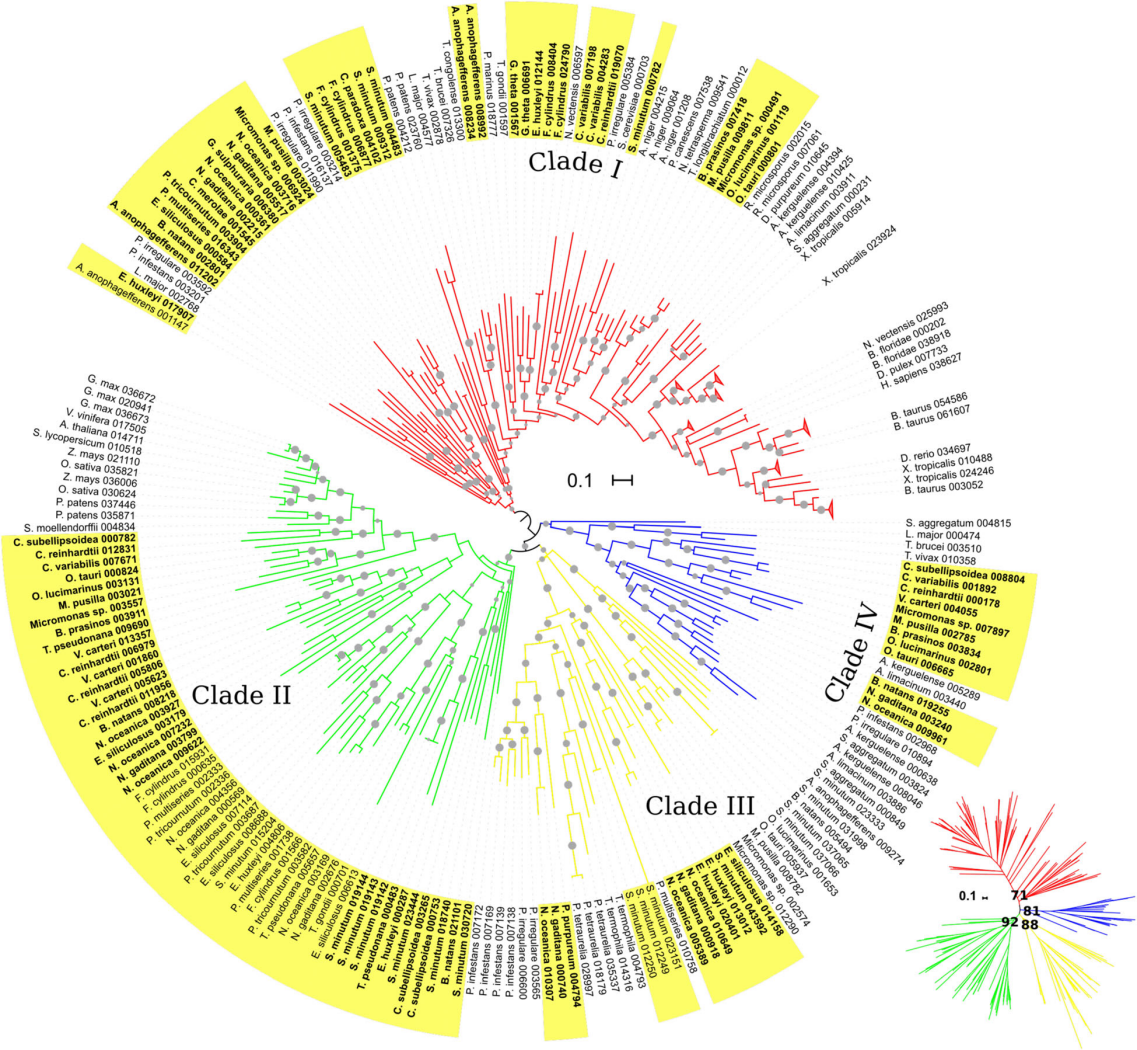
Phylogenetic relationships of algae DGAT2 sequences. Rooted circular phylogram representation of the tree generated by the maximum likelihood (ML) method (500 bootstraps) on the conserved regions of proteins from the DGAT2 family of the species detailed in Table 1 and Additional file 1. *Gray circles* represent ML bootstrap values > 50. Labels highlighted in *yellow* are algal sequences, numbers after the abbreviated species names are internal IDs. Due to high number of sequences, some subclades were collapsed to allow proper visualization of important leaves and branches. The *scale bar* represents 0.1 amino acid substitution per site. The inset is the same tree as an unrooted radial phylogram, with the ML bootstrap values for the four main clades: I-IV. To view the trimmed MSA used to reconstruct the tree in Phylip format and the raw consensus tree in Newick format, see Additional file 8

Phylogenetic analysis of all the full-length DGAT2s from algae and other eukaryotic taxa uncovered interesting facts. With the exception of a few well-supported orphans, DGAT2 proteins cluster in four main groups (Fig. 6). All the animal and fungal DGAT2 sequences cluster together, in what we called Clade I or the animal-type DGAT2 clade. All the algae higher ranks have sequences related to this clade, albeit in different subclades. The second group contains all the DGAT2 sequences from plants, and numerous members from most of the algae analyzed. We called this Clade II or the plant-type clade. Interestingly, this clade only contains sequences from photosynthetic eukaryotes, regardless of their taxonomic classification. The third clade consists exclusively of protein sequences from photosynthetic and non-photosynthetic SAR species, with the exception of two sequences from the haptophycean alga *Emiliania huxleyi*. Hence, we called this Clade III or the SAR-type DGAT2 group. Finally, the last clade contains several sequences from SAR, single members from most green algae and a few sequences from Excavata. We called this Clade IV. A sequence logo analysis of motifs 1–6 shows that, with the exception of a few residues, all the clades show a high level of conservation in the completely conserved amino acids (Fig. 5b). Regarding *C. reinhardtii* DGAT2, DGTT1 clusters in the animal-type clade, whereas DGTT2, DGTT3, DGTT4, and DGTT5 cluster in the plant-type clade, although in different subclades, similarly to what was previously reported [48]. The newly identified DGAT2, corresponding to gene ID Cre08.g377300, clusters in Clade IV. A majority of the proteins within this clade share the residue replacements present in the *C. reinhardtii* homolog (Fig. 5b). Most interestingly, a majority of the members of this clade contain a strikingly high number of predicted transmembrane domains (e.g. six for *C. reinhardtii*, seven for *B. prasinos*, nine for *C. variabilis*, eight for *V. carteri*), as opposed to the remaining three clades, which contain an average of two or three transmembrane segments, with very few sequences above this number (Additional file 4). This could point to important differences, worth exploring, in DGAT2-membrane association and catalysis.

### DGAT3 in-silico analyses

**Fig. 7:**
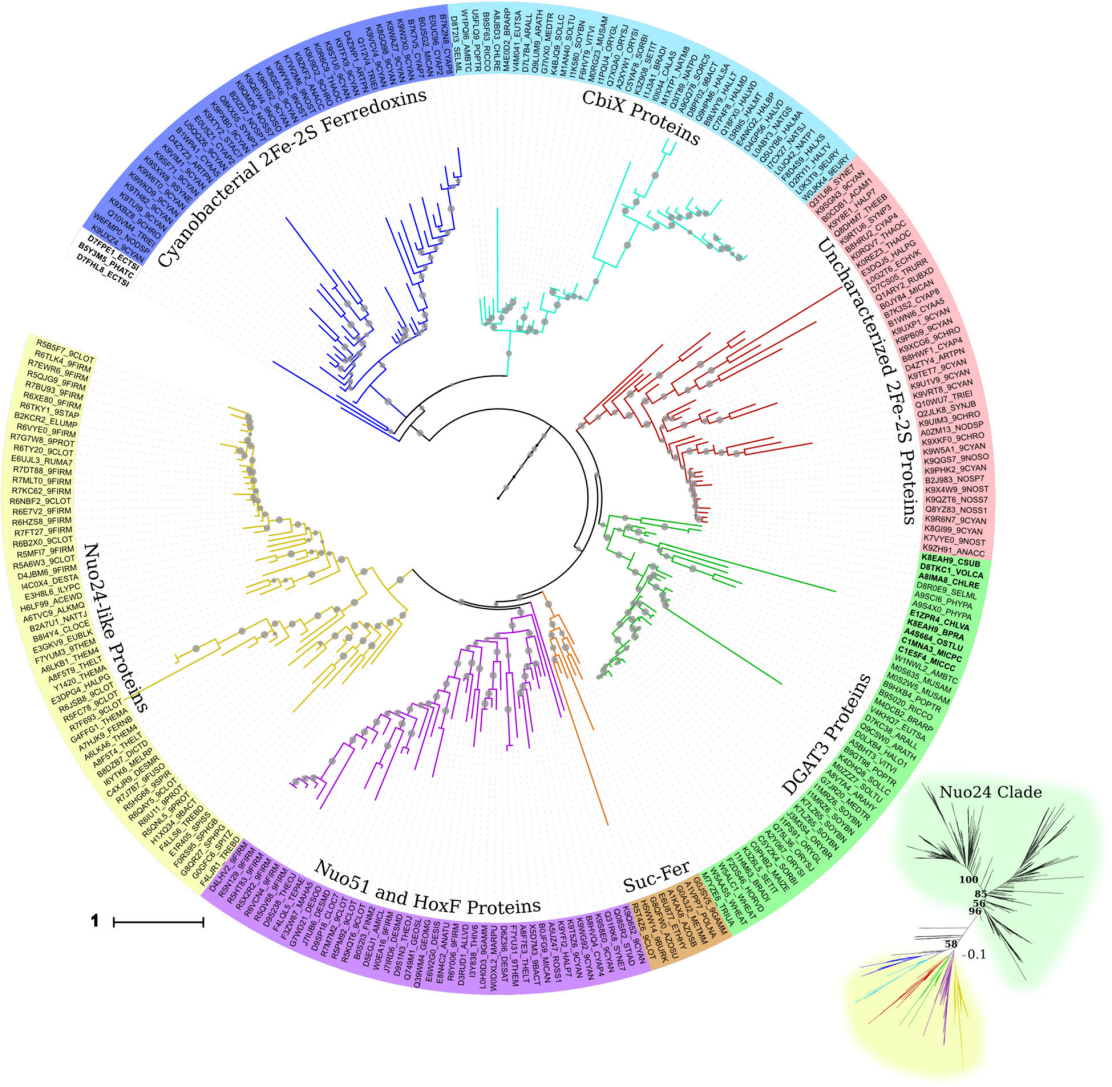
Phylogenetic relationships of algae thiorredoxin-like ferredoxin domains. Detailed rooted circular phylogram representation of the non Nuo24 clades of the tree generated by the maximum likelihood (ML) method (500 bootstraps) on the conserved regions of thiorredoxin-like ferredoxin domains of the species detailed in Table 1 and Additional file 1. Gray circles represent ML bootstrap values > 50. IDs correspond to Uniprot IDs. Labels in *bold* are algal sequences. Other sequences from species not included in Additional file 1 were also added to the analysis and are shown by their Uniprot IDs. The scale bar represents 0.1 amino acid substitution per site. The inset is an unrooted radial phylogram representation of the whole tree, which includes the Nuo24 clades, with the ML bootstrap values for the four main subgroups. To view the trimmed MSA used to reconstruct the tree in Phylip format and the raw consensus tree in Newick format, see Additional file 9. Suc-Fer, ferredoxin-like sucrases

One of the most remarkable findings of our data mining was the identification in green algae of sequences with moderate similarity to a soluble type of DGAT, DGAT3 (Table 2), to this date only characterized in a handful of higher plants, including *A. thaliana* [24], tung tree *(Vernicia fordii*) [40] and peanut (*Arachis hypogaea*) [39]. DGAT3 proteins are composed of an amino-terminal region containing several disordered stretches and a carboxy-terminal thioredoxin-like 2Fe-2S cluster-binding domain (2Fe-2S hereafter). When performing HMMER data mining starting with a profile hmm file containing all well-characterized plant DGAT3 proteins, many of the sequences identified with high scores were similar to DGAT3 only in their 2Fe-2S domain. The disordered amino terminal segments of many of these proteins produced MSAs with considerably high entropy that were not suitable for phylogenetic analysis. This prompted us to do a first phylogenetic reconstruction based on the relationships of the 2Fe-2S domain, which in pfam is known as the 2Fe-2S_thiored domain. The resulting ML tree (inset in Fig. 7) shows two main well-supported groups. The first major group consists of several subclades of the 24-kDa subunit of respiratory complex I NADH:ubiquinone oxidoreductase from both prokaryotes and eukaryotes (*nuoE* gene in *Escherichia coli*, *ndufv2* gene in humans). We refer to this clade as the Nuo24 clade. The other major group (shown in detail in Fig. 7) can be divided into several statistically supported clades of sequences with distinct putative catalytic activities: i) cyanobacterial 2Fe-2S thioredoxin-like ferredoxins, ii) bacterial and archaean CbiX proteins, a family of cobalt-chelatases that function in the anaerobic biosynthesis of cobalamin (vitamin B12) [50] and a group of related uncharacterized proteins from plants (streptophytes); iii) bacterial sucrases, proteins that resemble ferredoxin and appear to have sucrolytic activity [51]; iv) a group composed of both bacterial Nuo51 proteins (the 51-kDa subunit of respiratory complex I NADH:ubiquinone oxidoreductase) and several cyanobacterial hoxF proteins (a subunit within the diaphorase moiety of the reducing hydrogenase) [52] and v) a group of bacterial proteins distantly related to Nuo24.

The last monophyletic clade in Fig. 7 with moderate support (ML bootstrap support = 64) is composed of two subgroups. The first one comprises the thioredoxin-like 2Fe-2S domains from a group of uncharacterized proteins from cyanobacteria. Although some of the homologs within this group are reported as nucleic acid-binding proteins, this is mostly based on the presence of an OB fold, which might be involved in the binding to other molecules. The second group includes thioredoxin-like 2Fe-2S domains present in well-characterized DGAT3 proteins, such as the *A. thaliana* (Uniprot ID: Q9C5W0_ARATH) and *A. hypogaea* DGAT3 homologs (A8VTA4_ARAHY), as well as putative DGAT3 proteins from all the green algae included in the analysis. Interestingly, the DGAT3 group is composed exclusively of 2Fe-2S from Chloroplastida (plants and green algae), with no homologs identified in red algae or glaucophytes. The only exception was the clustering within this group of a thioredoxin-like 2Fe-2S domain of a protein from the Deltaproteobacteria *Haliangium ochraceum.* This domain was the only one within the DGAT3 clade that obtained a lower score when confronted to the DGAT3-specific hmm file than to the hmm files of several other clades within the tree. This, together with the long branch length of the corresponding leaf and the heterogeneity of its positioning when analyzing many of the compatible trees used by PhyML to build the consensus (data not shown), puts a question mark on the proper clustering of this sequence.

**Fig. 8:**
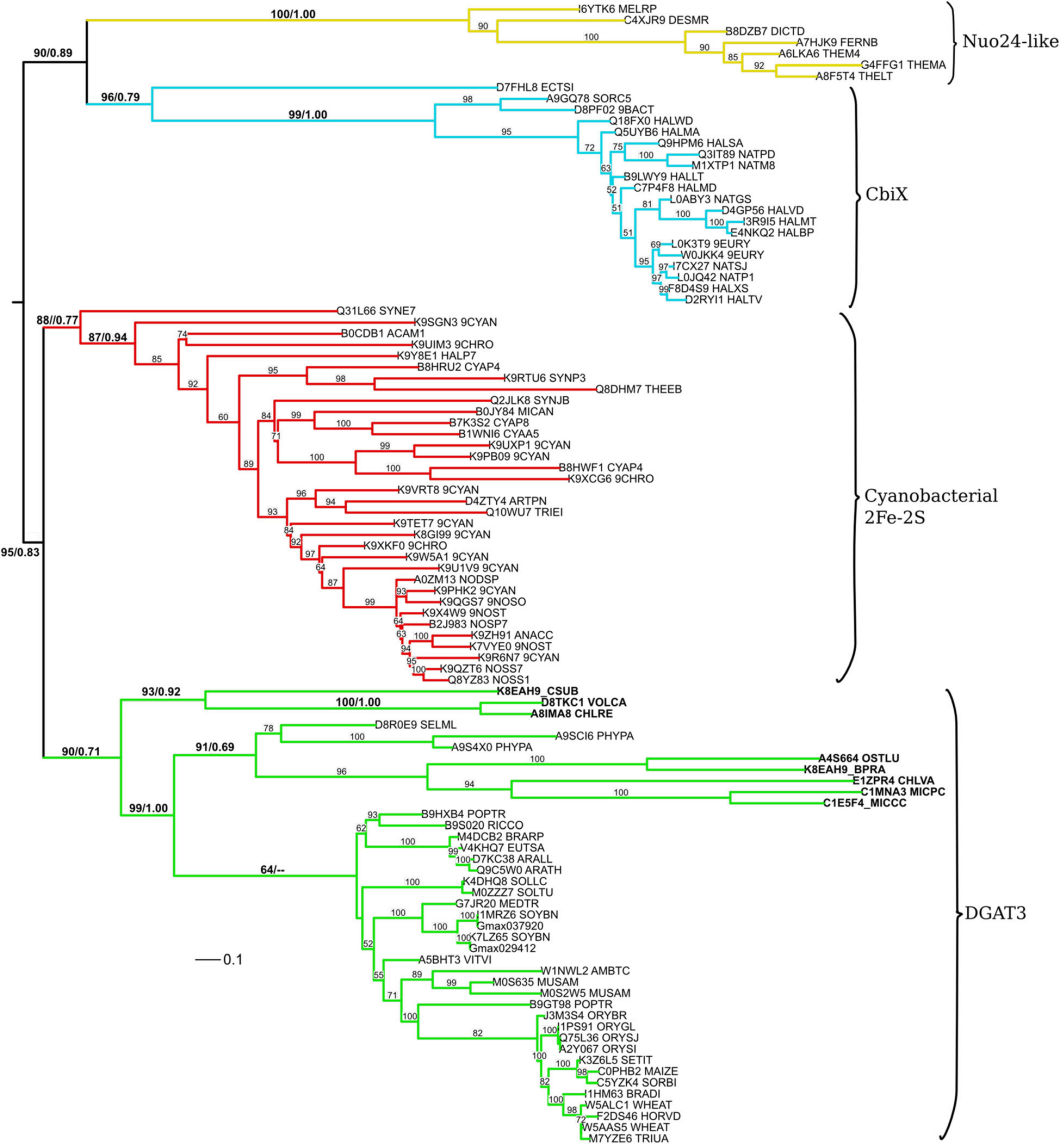
Phylogenetic relationships of algae DGAT3 sequences. Rooted phylogram representation of the tree generated by the maximum likelihood (ML, 1000 bootstraps) and Bayesian methods on the conserved regions of DGAT3 and related sequences. ML bootstrap values are shown for all leaves, Bayesian posterior probabilities are shown for the major branches after the bootstrap value, separated by a hash. One branch does not show the posterior probability because its value was below 0.50. Labels are Uniprot IDs, labels in *bold* correspond to algal sequences. The names and sequences within the four clades that were aligned to reconstruct the tree are shown with *brackets*. The *scale bar* represents 0.1 amino acid substitution per site. To view the trimmed MSA used to reconstruct the trees in Phylip format and the raw consensus ML and Bayesian trees in Newick format, see Additional file 10

When eliminating sequences that contributed to the high entropy of the full DGAT3 MSAs, we could establish phylogenetic relationships of DGAT3 sequences using a much larger portion of the proteins, which included most of the amino end. The resulting tree, shown in Fig. 8, shows that the DGAT3 clade is only composed of proteins from higher plants and green algae, with excellent support provided by both ML and bayesian analyses. In addition, this tree supports our findings that reveal that the DGAT3 group has a most recent common ancestor with a group of cyanobacterial 2Fe-2S proteins.

**Fig. 9:**
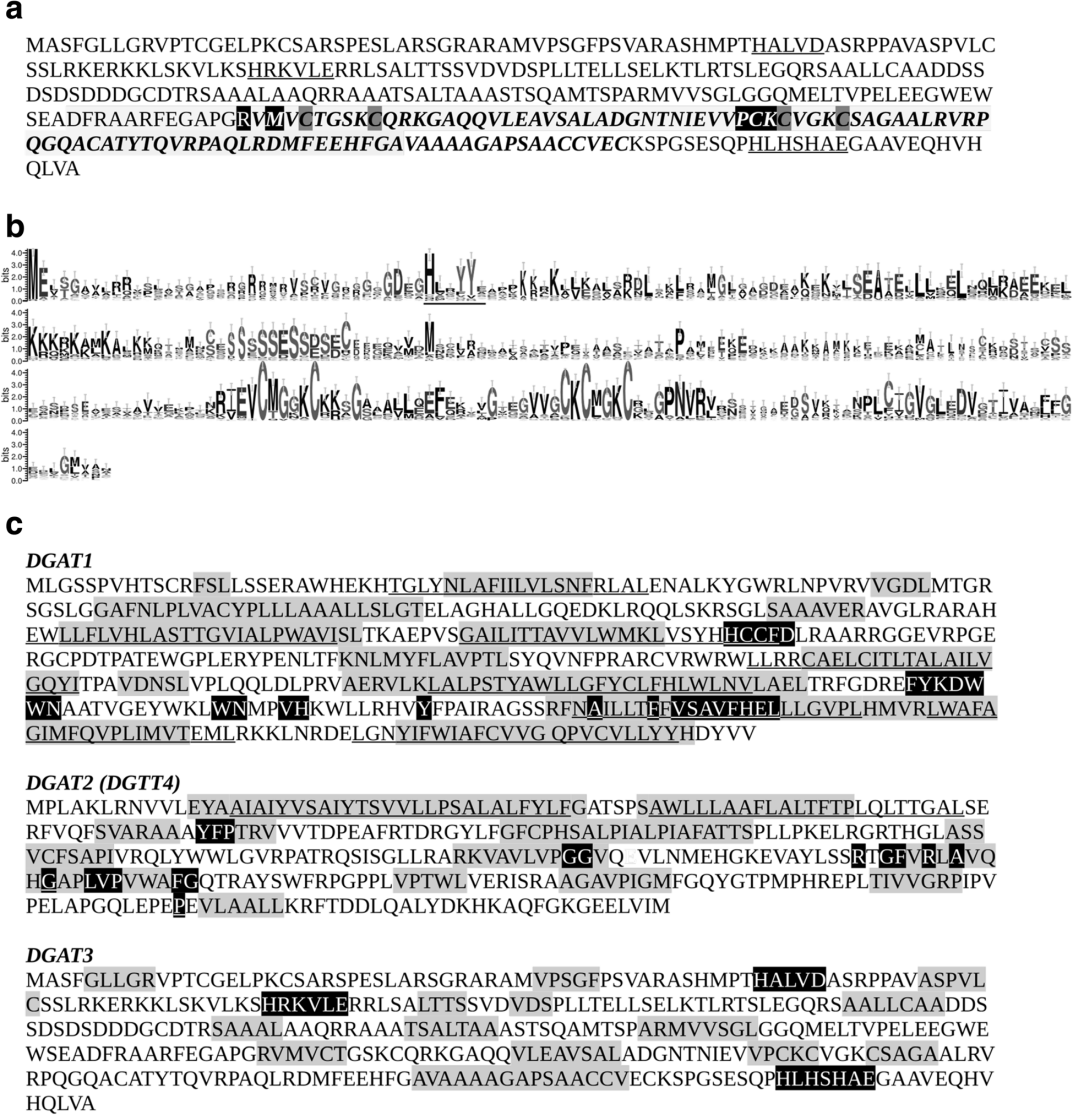
Sequence characteristics of DGAT3. **a** *C. reinhardtii* DGAT3 predicted amino acid sequence. Putative catalytic sites are underlined. *Highlighted* are the cysteines predicted to participate in 2Fe-2S binding (in *gray*) and the residues potentially involved in dimerization (in *black*). The *bolded italicized* residues correspond to the 91 amino acids modeled on the thioredoxin-like 2Fe-2S ferredoxin from *Aquifex aeolicus*. **b** Sequence logo of the DGAT3 family. The sequences within the DGAT3 clade of Fig. 8 were aligned, and the MSA was trimmed using BMGE to eliminate gaps (see Methods). The *y* axes show the logo bits; error bars are shown on each position. *Underlined* is the putative acyltransferase catalytic site that is most conserved amongst the members of the clade. **c** Comparison of *C. reinhardtii* DGAT1 partial sequence published by Boyle et al. [9], DGAT2 (DGTT4) and DGAT3 sequences. Highlighted in *gray* are the hydrophobic regions obtained by Kyte & Dolittle hydrophobicity scale analysis using a window size of 9. *Underlined regions* indicate transmembrane segments predicted by TM-HMM analysis. Residues highlighted in *black* show well established and/or putative conserved catalytic motifs

Since algal DGAT3 proteins have not been previously characterized, we decided to do a series of *in-silico* and empirical analyses with the purpose of obtaining further information about this emerging protein family. Figure 9a shows the amino acid sequence of *C. reihardtii* DGAT3. The carboxy-terminal region features the four cysteines involved in 2Fe-2S binding, as well as amino acids involved in dimerization of the polypeptide, as determined by amino acid similarity using delta-blast. Figure 9a also shows the 91 amino acids that could be modeled with a 99.8% confidence on the thioredoxin-like 2Fe-2S ferredoxin from *Aquifex aeolicus* (template d1f37b). In contrast, the formation of dimers could not be modeled with acceptable confidence by I-COTH, from the I-Tasser package. Secondary structure analysis predicted that *C. reinhardtii* DGAT3 is composed of 40% disordered regions, 47% of alpha helices and 9% of beta strands. Disordered regions are mainly concentrated at the amino terminus of the protein. The catalytic site of DGAT3 is not known for any of the members identified so far. However, the catalytic residues of many acytransferases have been determined, and a histidine (H) seems to be mandatory for activity in most of the protein families, often accompanied by an aspartic (D) or glutamic acid (E) separated by a few amino acids, most frequently four. In the DGAT1 and DGAT2 families, this motif is towards the carboxy end of the protein [29, 30], whereas it tends to be towards the middle portion of the protein or the amino end in GPAT and AGPAT [53]. We identified three motifs in *C. reinhardtii* DGAT3 that are similar to acyltransferase motifs, shown in Fig. 9a. Two of them are in the amino-terminal portion, whereas the other one is to the carboxy terminus of the protein, further to the carboxy end than the 2Fe-2S domain. It is noteworthy that the histidine in the motif closest to the amino end is conserved within the whole DGAT3 clade, as shown in the sequence logo in Fig. 9b.

Figure 9c and Additional file 5 show a hydrophobicity and transmembrane segment comparison between *C. reinhardtii* DGAT1, DGTT4 (a functionally characterized DGAT2,Cre03.g205050) and DGAT3. The three sequences showed a considerable percentage of hydrophobic regions (45, 40 and 25% for DGAT1, DGAT2 and DGAT3, respectively). Interestingly, in DGAT1 many of its hydrophobic regions translate into transmembrane segments, while only two hydrophobic regions represent transmembrane segments in DGAT2 and none in DGAT3. One of the most remarkable findings is that, in DGAT1 and DGAT2, the catalytic motifs either flank or are partially embedded in hydrophobic regions, while the putative catalytic motifs of *C. reinhardtii* DGAT3 are flanked by hydrophilic regions (Fig. 9c). This observation supports the idea that DGAT3 likely participates in a soluble TAG pathway. Nevertheless, the presence of hydrophobic regions in *C. reinhardtii* DGAT3 is indicative of both protein membrane association and the interaction with lipid substrates.

We also did subcellular localization prediction analyses of DGAT3 proteins. Table 2 shows that the DGAT3 proteins from most of the green algae analyzed, with the exception of the homolog from *Coccomyxa subellipsoidea*, were predicted to be localized to the chloroplast using PredAlgo, an algorithm specifically trained with green algal proteins. The *Ostreococcus tauri* DGAT3 has a truncated amino end due to several unidentified amino acids, which hinders prediction. Although TAGs have been shown to accumulate in the chloroplast under nitrogen starvation [54, 55] and light stress [56], a *de novo* pathway for the synthesis of TAGs within this organelle has not been reported yet. In order to extend the results obtained with DGAT3, we did in-silico analysis of protein targeting for all the DGATs and PDATs identified in algae. The results (Table 2) show that some of the DGAT1, DGAT2 and PDAT homologs from green algae are predicted to be targeted to the chloroplast, whereas we did not find any cases of predicted chloroplast localization for any of these enzymes in SAR, haptophycean and cryptophycean algae. For *C. reinhardtii* in particular, we analyzed the complete *de novo* pathway starting from the activation of fatty acids. Table 3 shows that, with the exception of phosphadidate phosphatase (PAP), all the enzymatic steps of the pathway have at least one isoform with evidence of expression either at the transcriptional or protein level and predicted chloroplast targeting. Our analysis also revealed that this pathway could be soluble, carried out by enzymes associated to chloroplast membranes but with no transmembrane segments (Table 3). This would be consistent with the soluble, albeit cytosolic, pathway proposed in developing cotyledons of peanut, in which DGAT3 is involved [39].

**Table 3:** *C. reinhardtii* sequences within the TAG pathway with predicted chloroplast targeting

Phytozome v11.0 ID	Length (aa)	PredAlgo	Membrane (M)/Soluble (S)	Evidence^a^
LACS				
Cre12.g500715.t1.2	1242	C	S	Transcript level
Cre06.g299800.t1.2	784	C	M	Transcript level
GPAT				
g6130.t1	456	C	M	Protein Level
Cre02.g143000.t1.2	410	C	S	Protein Level
GPAT/LPAT				
Cre05.g248150.t1.2	370	C	M	Transcript level
Cre17.g738350.t1.2	325	C	M	Transcript level
DGAT1				
Cre01.g045903.t1.1	464	C	M	Transcript level
DGAT3				
Cre06.g310200	346	C	S	Transcript level
PDAT				
Cre02.g106400	1040	C	M	Protein level

^a^ Transcript level refers to evidence of existence by EST tagging, RNAseq, Microarray or RT-PCR analyses. Protein level refers to evidence by proteomics

### Characterization of *C. reinhardtii* DGAT3 enzymatic activity

**Fig. 10:**
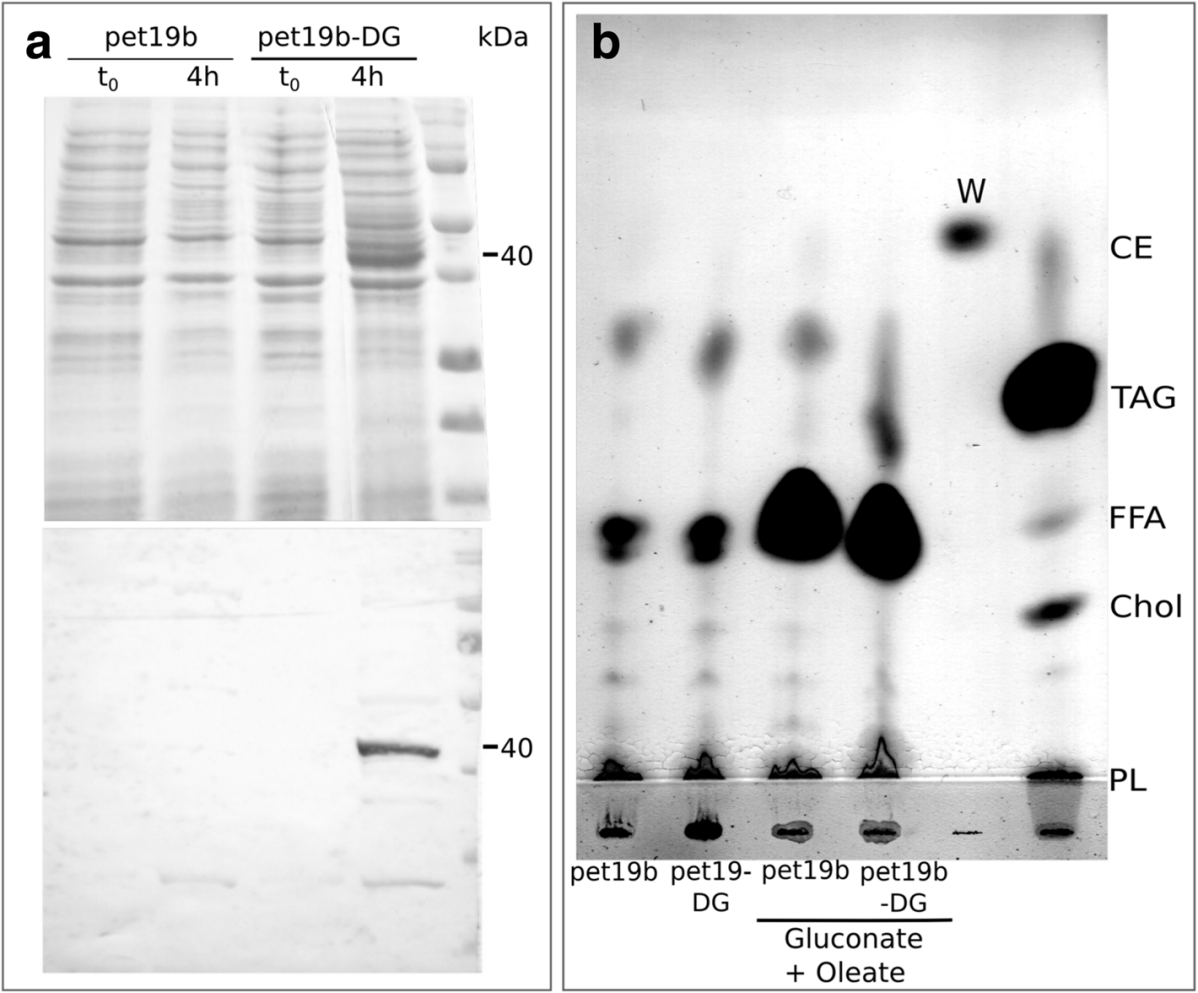
Characterization of *C. reinhardtii* DGAT3 expressed in *E. coli*. The coding sequence of *C. reinhardtii dgat3* was cloned into pet19b vector (pet19b-DG) and transformed into *E coli* cells. The empty vector (pet19b) was transformed as a control. Protein induction was done on both DGAT3-expressing and control cells. Samples were taken before (t_0_) and 4 h after induction (4 h). **a** Soluble proteins from both samples were analyzed by 12% SDS-PAGE and either stained with Coomassie brilliant blue (*upper panel*) or transferred to nitrocellulose (*bottom panel*). The membranes were incubated with rabbit anti-*C. reinhardtii* DGAT3 polyclonal antisera. The protein standards are shown on the *right lanes*, the *dashes* indicate the extra band in the gel upon induction and the protein recognized by the antisera, of approximately 40 kDa. **b** Lipids from *E. coli* cells were extracted with methanol: chloroform (1:1) and analyzed by thin layer chromatography (TLC) using a mix of hexane-diethyl ether-formic acid as the solvent system. Standards are: CE, chloesterol ester; W, wax ester; TAG, triglyceride; FFA, free fatty acids; Chol, cholesterol; PL, phospholipids

For DGAT families with well characterized members, such as DGAT1 and DGAT2, it is very common to infer activity of a new member by simple similarity analysis. However, DGAT3 is a very incipient family, with very few members characterized. In addition, the DGAT3 family is completely unrelated to DGAT1 and DGAT2, and the conserved sites that are important for activity are not known. With the purpose of interrogating *C. reinhardtii* DGAT3 catalytic activity, we expressed this protein in *E. coli* cells (Fig. 10a). Most groups of bacteria, including *E. coli*, do not accumulate TAGs to significant levels, which allows to easily detect the presence of such compounds dependent on the expression of a heterologous enzyme. TLC analysis of *E. coli* total lipids showed that *C. reinhardtii* DGAT3 expression produced an increase in the accumulation of TAGs compared to the control (Fig. 10b). Interestingly, the TAG spot was only visible when gluconate and oleate were added to the culture during induction. The addition of those compounds triggers in the bacterial cell a set of responses collectively known as *storage condition,* which was previously used with the purpose of driving the bacterial metabolism towards TAG synthesis [57, 58]. The fact that TAGs were not detectable in DGAT3-expressing *E. coli* cells without the addition of an excess of substrate (oleate) might indicate that, in a prokaryotic system, DGAT3 activity cannot compete with phospholipid synthesis, which is a major lipid pathway in *E. coli.* Interestingly, a spot that migrated further than the TAGs appeared in all samples of the TLC. According to its positioning relative to the standards and to results reported by others [59, 60], this spot might correspond to quinones, a type of neutral lipid present in *E. coli* that can be co-extracted with TAGs [58]. Future experiments will be aimed at characterizing the DGAT3 activity further and at determining the precise identity of the additional spot in TLCs.

## Discussion

### Data-mining and phylogenetic analyses

In this work, we have done a thorough data mining of the TAG pathway, with an emphasis on the phylogenetic connections between homologs of algae from distinct supergroups. We have unveiled interesting relationships of the enzymes involved in the committing steps, DGAT and PDAT, that might have important evolutionary and functional implications. Our data mining and phylogenetic analyses allowed us to make testable predictions on the identity of enzymes that are part of complex superfamilies. Future experimentation will put to the test the hypotheses arisen from this work.

One of the most remarkable findings of our data mining was the identification of sequences with similarity to plant soluble DGAT3 proteins, exclusively in the green algal lineage. There are several interesting facts about DGAT3. First, phylogenetic analysis of either the 2Fe-2S domain or a much larger portion of the protein show that the DGAT3 clade shares a most recent ancestor with a group of uncharacterized proteins from cyanobacteria. The highest HMMER search scores of the DGAT3 hmm file were, after the members of the DGAT3 clade, obtained with cyanobacterial proteins. In contrast, no prokaryotic sequences were above the inclusion threshold in the searches done for DGAT1, DGAT2 and PDAT, hence no prokaryotic sequences are included in the phylogenetic trees of these protein superfamilies. These findings suggest that DGAT3 is a very distinctive protein within the TAG-synthesizing pathways. DGAT1, DGAT2 and PDAT are proteins exclusive to eukaryotes, in agreement with the fact that the synthesis of TAGs is widespread in eukaryotic organisms, whereas TAGs accumulate to significant levels only in a few prokaryotic groups [61, 62]. These enzyme superfamilies most likely evolved in eukaryotes coincident with an increase in the complexity of the lipid metabolic pathways, as exemplified by the intricate phylogenetic relationships between DGAT1 and SOAT, or PDAT and LCAT or by the extensive multiplication of DGAT2 in algae. DGAT3, in contrast, is likely a relic of cyanobacterial ancestry.

Plant DGAT3 proteins are reported to be cytosolic, in agreement with their localization prediction. In contrast, most green algal DGAT3 are predicted to be targeted to the chloroplast. We could speculate that the members of Chloroplastida (plants and green algae) inherited DGAT3 from cyanobacteria and, if the localization predictions are confirmed, this protein remained chloroplastic in green algae, whereas it found its eukaryotic functions in the cytosol in higher plants. This would point to another interesting difference between green algae and higher plants regarding TAG regulation. We could further question whether DGAT3 was acquired by the whole Archaeplastida supergroup during primary endosymbiosis and subsequently lost in red algae and glaucophytes, or evolved later on only in Chloroplastida. Although the latter seems the most parsimonious explanation, further analyses could certainly allow to differentiate between these two possibilities. Regardless of its origin, DGAT3 seems to be exclusive to the green lineage. Proteins with this characteristic have been clustered within the ViridiCut2 group in a recent inventory of proteins common to plants and green algae [63]. The authors of that analysis determined that most of the ViridiCut2 proteins lack cyanobacterial homologs and might be involved in eukaryotic processes that are not exclusively associated with the photosynthetic function. It is difficult to unambiguously answer whether DGAT3 lacks cyanobacterial homologs, since the function of the members of the cyanobacterial clade related to DGAT3 is not known. Nevertheless, the fact that TAG synthesis is mostly a eukaryotic function that, although related to the plastid, exceeds photosynthesis, suggests that DGAT3 shares this characteristic with other ViridiCut2 proteins.

### Characteristics of *C. reinhardtii* DGAT3

DGAT3 proteins are characterized by the presence of an 2Fe-2S domain that has a fold similar to that present in thioredoxins [64]. Many of the bacterial proteins containing this domain are small (ca. 80–100 amino acids), soluble low-potential electron carriers (between −0.2 and −0.45 V) with a single 2Fe-2S cluster [65]. The exact role of many of these proteins is still unclear. Their homologous domains in larger redox enzymes (such as Nuo24, Nuo51 and NAD-reducing hydrogenases) function as electron carriers [66, 67]. In this scenario, we could imagine that DGAT3 has a dual activity in which electron transfer is coupled to the acylation reaction. Although the precise site for catalytic activity of DGAT3 proteins is not known, there are few important amino acids that seem to be common to many acyltransferases, as analyzed by Saha et al. [39]. We identified three motifs that could catalyze acylation reactions in *C. reinhardtii* DGAT3, two in the amino terminal region and one to the carboxy-terminal portion of the 2Fe-2S cluster-binding domain. Future experimentation will shed light to the precise site in DGAT3 where catalysis occurs.

### The chloroplast TAG pathway and possible involvements of PDAT, DGAT1 and DGAT3

The importance of a chloroplast pathway for the synthesis of TAGs in green algae became evident with recent work done in *C. reinhardtii*. Fan et al. [54] showed that, during nitrogen starvation, a high proportion of the TAG produced is composed of 16-carbon (C16) fatty acids in the sn-2 position of the glycerol backbone and is, in fact, mostly synthesized from DAG generated in the chloroplast. The presence of lipid droplets inside the chloroplast was also evidenced by microscopy in starch-less *C. reinhardtii* mutants grown in mixotrophic conditions [54, 55, 68]. One major player in the synthesis of chloroplast TAGs in green algae could be PDAT. The *C. reinhardtii* PDAT homolog is known to localize to the chloroplast [69] and to use preferentially C16 fatty acids [70]. A loss-of-function analysis of *C. reinhardtii* PDAT revealed that it might contribute to approximately 25% of the TAG accumulation under nitrogen starvation in *C. reinhardtii* [9]. In contrast, it has been proposed that the contribution of the chloroplast to total TAG synthesis during nitrogen depletion could be much higher [54], suggesting that other mechanisms are involved. Analysis of chloroplast plastoglobuli and cytosolic lipid droplets in the green alga *Dunaliella bardawil* revealed similar fatty acid composition in the sn-2 and sn-1 + 3 positions for the TAGs in both types of lipid bodies, suggesting a common origin [71]. Under nitrogen starvation, the formation of cytosolic lipid droplets preceded that of plastoglobuli and appeared to be synthesized via the *de novo* pathway, whereas that of plastoglobuli seemed to be mostly originated from membrane remodeling [71]. Electron microscopy analysis revealed that the ER membrane and the cytosolic lipid droplets *per se* attach to the chloroplast envelope, leading the authors to hypothesize that cytosolic TAGs might be transferred to the chloroplast through associations between the ER membrane and the outer membrane of the chloroplast envelope (OMCE) [71]. Such associations were also observed in *C. reinhardtii* [55]. Therefore, despite the differences in TAG accumulation between the two algae, the idea that interactions between the ER and the OMCE allow the interchange of fatty acids, DAG and TAG between both compartments is likely for all green algae.

In *A. thaliana*, rosette lipid analysis revealed that TAG accumulation increases considerably under certain situations, such as ozone-related stress [72] or senescence [73]. In those conditions, TAGs accumulate preferentially in the chloroplast. Rosette leaf mRNA analysis evidenced that one clone annotated as DGAT1 increased notoriously in senescent leaves compared to young leaves, allowing the authors to propose that DGAT1 has an important role in senescence by sequestering de-sterified fatty acids coming from the thylakoid [73]. The presence of DGAT activity related to plant plastids was shown more than 30 years ago in isolated spinach chloroplasts, where it was found in the chloroplast envelope [74]. Unfortunately, back then, the isoform responsible for activity was not analyzed or identified, but it could be a DGAT1. In this work, localization analysis predicted that *C. reinhardtii* DGAT1 localizes to the chloroplast. In this scenario, and considering that this protein has transmembrane domains, it is possible that DGAT1 associates not only to the ER, but also to the OMCE, in *C. reinhardtii*.

In this context, the participation of DGAT3 could be completely different. Being a soluble, yet membrane-associated, protein with an 2Fe-2S domain, we could imagine a participation in the stroma, in relationship to the thylakoid membrane. Considering that it might be, the same as its homologous proteins characterized so far, a low potential redox protein, we could hypothesize that *C. reinhardtii* DGAT3 accepts electrons from the final transporters of the photosynthetic electron transport chain and reduces NADP+. The NADPH formed in this way could be used for fatty acid synthesis, which could then be used for TAG synthesis by DGAT3. Furthermore, DGAT3 might be responsible for TAG formation in the chloroplast in situations that produce an excess of electrons. The hypothesis that TAGs might serve as a sink for electrons moving through the photosynthetic electron transport chain has been previously postulated [1]. As previously suggested [75], during stress, excess photosynthetic electrons could be used to generate reducing equivalents, in the form of NADPH, for fatty acid synthesis. Since an excess of free fatty acids is potentially harmful itself, the dual action of DGAT3 would ensure the coupling of NADP+ reduction, fatty acid synthesis and fatty acid acylation into TAG, providing a complete protective mechanism. Although, during stress, much of this could occur in the ER as proposed [75], we hypothesize that the green algal chloroplast might have a complete pathway to do this in an independent fashion.

## Conclusions

To our knowledge this study represents, to date, the most comprehensive sequence analysis of the main enzymes that participate in TAG synthesis in algae, including their phylogenetic relationships with homologs from other organisms and important structure-function predictions. Our results confirmed the complex connections between the distinct acyltransferase superfamilies. Regarding the green alga *C. reinhardtii,* we observed that TAG synthesis is well represented by the presence of numerous acyltransferases, including one PDAT, one DGAT1, six DGAT2 and one DGAT3. DGAT3 is a soluble protein exclusive to the green linage, with predicted chloroplast localization. This protein is the only acyltransferase activity of those analyzed associated to a cyanobacterial heritage. Heterologous expression confirmed that *C. reinhardtii* DGAT3 is indeed a DGAT. As evidenced by subcellular targeting prediction, green algae are likely the only algal group with a chloroplast pathway for TAG synthesis. Methodologically, our analyses allowed us to conclude that data-mining by means of HMMER iterative search followed by phylogenetic analysis and/or protein clustering is a more adequate strategy for lipid functional genome analysis compared to the use of non-iterative heuristic methods, particularly for paraphyletic groups like algae.

## Methods

### Proteomes and databases used for data mining

Protein models from fully-annotated genomes (hereafter called complete proteomes, according to http://www.uniprot.org/keywords/KW-0181) [76] of 26 eukaryotic algae groups, supplemented with the complete proteomes of 53 non-algal eukaryotic organisms were used for sequence mining (all available by December 2015). Additional file 1 lists the eukaryotic species analyzed, their internal codes, the source of the proteome sequences and the corresponding references or sequencing projects. We followed taxonomic relationships between taxa according to Adl et al. [13]. Prokaryotic homologs were extracted from Reference Proteomes (EMBL-EBI, http://www.ebi.ac.uk/).

### Identification and analysis of algae homologs of the TAG synthesis pathway

Sequences were identified by HMMER version 3 [77] iterative profiling starting from seed multiple sequence alignments (MSAs). Seed MSAs were constructed with MAFFT at http://mafft.cbrc.jp/alignment/server/index.html [78] or hmmalign [77], using reviewed sequences obtained from Swissprot [76]. Seed MSAs were visualized and manually corrected using Aliview [79] and GeneDoc [80] and subsequently used to generate position-specific scoring tables (Hidden Markov Models, hmm files) using the hmmbuild tool from the HMMER suite. The models generated were used to search a compiled fasta file containing all the complete proteomes of the selected eukaryotic species, using the hmmsearch tool. Sequences above the default inclusion threshold (E-value = 0.01) were retrieved and sequences showing 100% identity were eliminated using CD-HIT [81]. The remaining sequences were aligned using MAFFT. The resulting MSAs were manually corrected. This mainly involved the elimination of sequences that did not have the hallmarks specific for each protein, used for scrutiny, and re-alignment of incorrectly aligned residues. The edited MSAs were used for the generation of new hmm files, re-starting the whole cycle. This process, for each protein, was repeated until convergence, at which point no new information was obtained in a new data-mining cycle. In order to ensure that we obtained the most complete datasets possible, the sequences from each phylogenetic group were retrieved and used in group-specific data-mining following the same procedure as the one described above. In order to analyze algae homologs in a true phylogenetic context, the seed and final hmm files were used to search for prokaryotic homologs. This was done by searching (using hmmsearch) the Reference Proteomes databases at the HMMER website, restricted to Bacteria and Archaea. All non-identical prokaryotic sequences better than a established inclusion threshold (E-value = 10^−8^) were added to the analysis.

In order to use only phylogenetically informative regions for the reconstruction of phylogenetic trees, final MSAs were trimmed using BMGE (Block Mapping and Gathering with Entropy) [82]. BMGE optional arguments were determined based on the conservation of secondary structure elements and were: −m BLOSUM30 -h 0.7 -g 0.4:0.2 -b 3. This procedure eliminated not only alignment columns according to entropy and gap content, but also eliminated sequences that contained a gap proportion higher than 40%. All the putative algae sequences that were eliminated using this criteria were confronted with the group-specific hmm profiles mentioned in the previous section, and the closest phylogenetic group was determined by the highest HMMER scores. Sequence logos were generated on desired regions of trimmed alignments using WebLogo 3 (http://weblogo.threeplusone.com/create.cgi) [83] with default settings and the Chemistry color scheme.

### Phylogenetic tree reconstruction

Maximum Likelihood and Bayesian methods were used for phylogenetic tree reconstruction. For Maximum Likelihood, PhyML 3.0 was used with default settings and 500–1000 bootstraps depending on the MSA [84]. IQ-TREE in ultrafast mode [85, 86] with defaults settings was used to obtain a fast consensus ML tree used as a starting tree in bayesian phylogeny reconstruction. For Bayesian analysis, we used Mr. Bayes 3.2.3 [87] with the previously mentioned starting tree with five perturbations and the WAG model, with its own default priors and invariant gamma distribution approximated with four substitutions. Two Metropolis-coupled Markov Chain Monte Carlo (MCMCMC) runs with four chains each and a temperature of T = 0.05 were done. Convergence of the chains in the Bayesian analysis was assessed by monitoring that the standard deviation of split frequencies was < 0.01 and with AWTY online, a system for the graphical exploration of MCMC convergence in Bayesian phylogenetic inference (http://king2.scs.fsu.edu/CEBProjects/awty/awty_start.php) [88]. Trees were visualized with Dendroscope v3.2.10 [89] and final tree editing was done using iTOL [90].

### Protein localization predictions

Sub-cellular localization prediction was done using both manual and automated methods: PredAlgo (https://giavap-genomes.ibpc.fr/cgi-bin/predalgodb.perl?page=main) was used for the prediction of transit peptides in Archaeplastida algae [91], Hectar (http://webtools.sb-roscoff.fr/tool_runner?tool_id=toolsheddev.sb-roscoff.fr%2Frepos%2Flgueguen%2Fhectar_3_0%2Fabims_hectar%2F1.0) was used for Heterokonts [92]. In addition, a manual method was used for organisms containing secondary plastids, as described elsewhere [93, 94]. Briefly, sequences were first analyzed using SignalP (http://www.cbs.dtu.dk/services/SignalP/) [95]. For those sequences with positive prediction of endoplasmic reticulum (ER) trafficking, the signal peptides were manually removed and the resulting sequences were further analyzed using TargetP [95] to test for the presence of plastid targeting peptides.

### Structural predictions

Important amino acids in *C. reinhardtii* DGAT3 were determined from the literature [25, 39] as well as via blastp, using the delta-blast algorithm in the NCBI website (https://blast.ncbi.nlm.nih.gov/Blast.cgi). *C. reinhardtii* DGAT3 three-dimensional (3D) modeling and secondary structure prediction were done using Phyre2 [96] and I-Tasser [97] with default settings. Hydrophobicity analyses of *C. reinhardtii* DGAT1, DGAT2 and DGAT3 were done with ProtScale from the ExPASy web server [98] and the Kyte & Doolittle amino acid hydropathicity scale [99] with a window size of nine for hydrophobic region identification. Transmembrane segment analysis was done using TMHMM v2.2 [100].

### Expression of *C. reinhardtii* DGAT3 in *Escherichia coli*

The *C. reinhardtii* DGAT3 coding region was amplified by PCR using the following oligonucleotides: *dgat3 Forward* 5' CATATGGCCTCTTTCGGCCTGCTGG 3' and *dgat3 Reverse* 5' GGATCCTTACGCCACTAGCTGATGC 3'. PCR products were cloned into Zero Blunt® TOPO® PCR Cloning Kit (Life Technologies, Carlsbad, CA, USA), further subcloned into pET19b (Novagen, Madison, WI, USA) and transformed into *Escherichia coli* BL21. Protein induction was done according to the Novagen pET system manual. Briefly, 500 μl of overnight *E. coli* cultures (grown in LB with 100 μg ml^−1^ ampicillin at 28 °C) were added to fresh LB with the same antibiotic. Cultures were grown at 37 °C to an OD of 0.6 and induced with 1 mM IPTG for 4 h. Cells were harvested by centrifugation at 10,000 × g for 20 min. For lipid analysis, 0.1% sodium oleate and 0.1% sodium gluconate were added to the induced cells 4 h after IPTG addition.

### Generation of anti-*C. reinhardtii* DGAT3 polyclonal antisera

Histidine-tagged *C. reinhardtii* DGAT3 was purified using Ni-NTA agarose according to the manufacturer’s instructions (QIA expressionist; Qiagen, Valencia, CA, USA). Briefly, cells were lysed by sonication in lysis buffer (20 mM Tris-HCl pH 8, 0.5 M NaCl, 5 mM imidazole, 1 mM PMSF) and cell debris were eliminated by centrifugation at 10,000 × g for 20 min. Supernatants were batch-incubated with the Ni-NTA resin for 1 h at 4 °C and the slurry was transferred to a plastic column. Three consecutive washes with lysis buffer were done with the following imidazole concentrations: 5, 20 and 60 mM. The 6xHis-DGAT3 protein was eluted in lysis buffer at 100 mM imidazole, concentrated to 1 mg/ml and dialyzed into physiological saline solution (0.85% NaCl) to generate rabbit polyclonal antibodies. To this aim, two rabbits were immunized subcutaneously with 500 ug of protein mixed with equal volumen of complete Freund's adjuvant on days 0, 7, 16 and with incomplete adjuvant on day 25 and 35. Afterwards, the animals were anesthetized and sacrificed. The immune sera were frozen at −20 °C and used for DGAT3 detection. All the procedures were performed according to the local institutional animal care (CICUAL, Universidad de Buenos Aires, Argentina).

### Protein electrophoresis and Western Blot analysis

For protein preparations, *E. coli* cells were sonicated and centrifuged at 10,000 × g for 20 min. Proteins in the supernatant were quantified using the bisinconinic acid reagent (Sigma, St. Louis, MO), separated by 12% SDS-PAGE and transferred to nitrocellulose (Genesee Scientific, San Diego, CA). Rabbit polyclonal antisera against *C. reinhardtii* DGAT3 were incubated overnight at 4 °C. Alkaline phosphatase-labeled anti-rabbit secondary antibodies were used. Protein gel blots were developed using nitroblue tetrazolium and 5-bromo-4-chloro-3-indolyl phosphate (Sigma).

### Lipid extraction and thin layer chromatography analysis

For lipid analysis, cell pellets were sonicated and extracted with methanol:chloroform (1:1). Thin layer chromatography (TLC) analysis was done in silica plates with a neutral lipid solvent system (hexane:diethyl ether:formic acid, 80:20: 2), loading the volume of lipids corresponding to 2.5 mg of protein. A mix of wax esters and total lipid extract from rat liver were used as standards. Plates were developed using the sulfuric acid charring method. Briefly, plates were sprayed with a 8% CuSO_4_ (w/v), 10% H_3_PO_4_ (v/v) solution and the lipids visualized as black carbon depots formed after heating the plates at 180 °C for 1 h.

## Additional files

Additional file 1: Sources of the proteome files used for TAG pathway biocomputational HHMER iterative profiling. (XLSX 20 kb)
Additional file 2: Number of LCAT superfamily homologous sequences identified in algae by HMMER iterative data mining. (XLSX 8 kb)
Additional file 3: Full-length DGAT2 sequences identified in the selected algal species. (XLSX 10 kb)
Additional file 4: Transmembrane segment prediction of algal DGAT2 sequences. (PDF 1069 kb)
Additional file 5: Hydrophobicity and transmembrane segment analysis of *C. reinhardtii* DGATs. (PDF 418 kb)
Additional file 6: Phylogenetic analysis of the LCAT superfamily in algae and other representative taxa. (TXT 49 kb)
Additional file 7: Phylogenetic analysis of the MBOAT superfamily in algae and other representative taxa. (TXT 68 kb)
Additional file 8: Phylogenetic analysis of the DGAT2 family in algae and other representative taxa. (TXT 63 kb)
Additional file 9: Phylogenetic analysis of thiorredoxin-like ferredoxin domains in algae and other representative taxa. (TXT 101 kb)
Additional file 10: Phylogenetic analysis of DGAT3 and related proteins in algae and other representative taxa. (TXT 24 kb)
