# Supplementary material for: Analysis of triglyceride synthesis unveils a green algal soluble diacylglycerol acyltransferase and provides clues to potential enzymatic components of the chloroplast pathway
Source: BMC Genomics. 2017 Mar 9;18:223. doi: 10.1186/s12864-017-3602-0 (PMC5343412; doi:10.1186/s12864-017-3602-0)
Supplement: Additional file 4: — Transmembrane segment prediction of algal DGAT2 sequences. (PDF 1069 kb) [file 12864_2017_3602_MOESM4_ESM.pdf]

DGAT2 Clade I

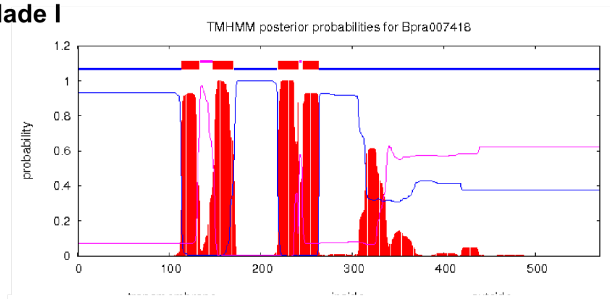

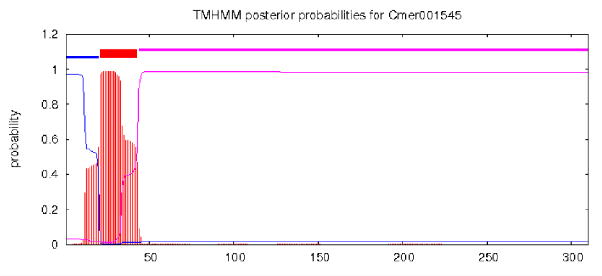

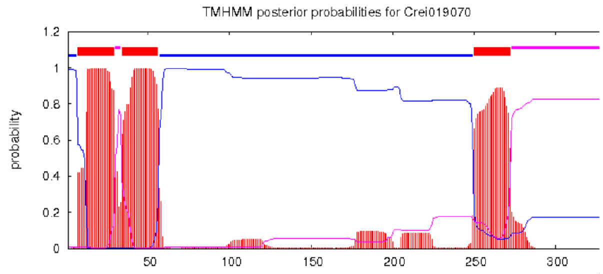

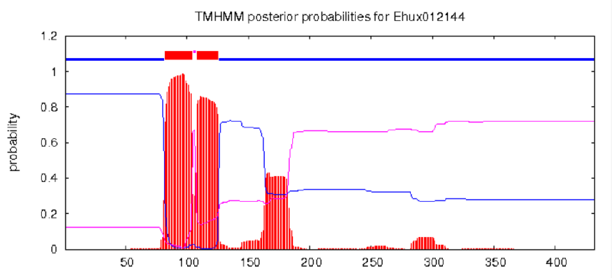

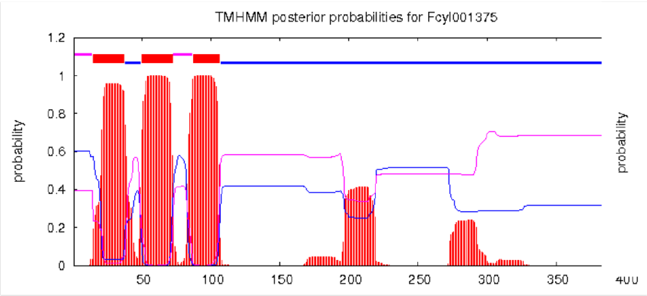

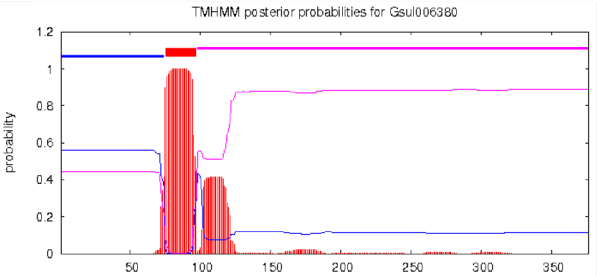

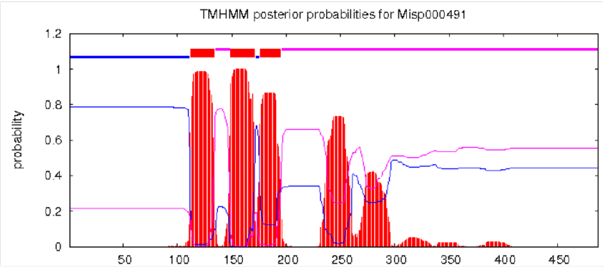

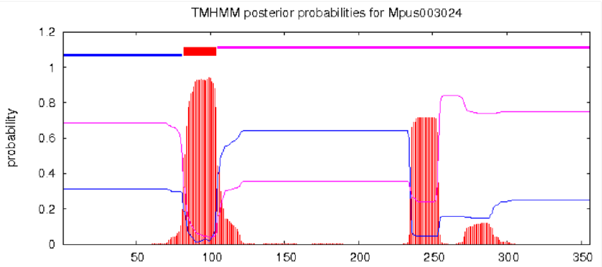

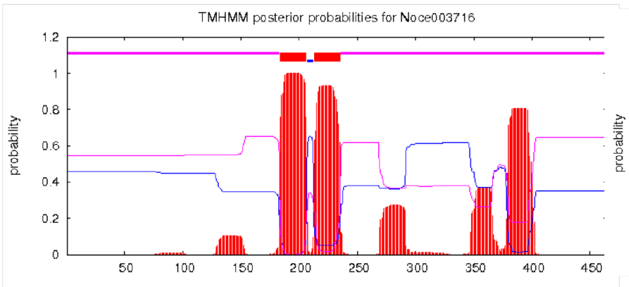

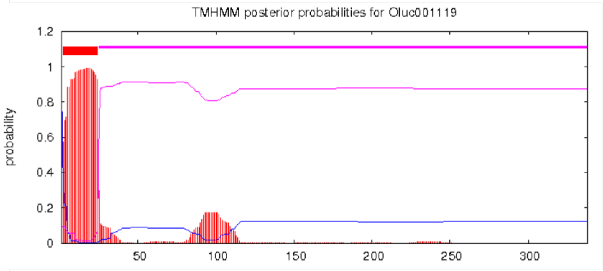

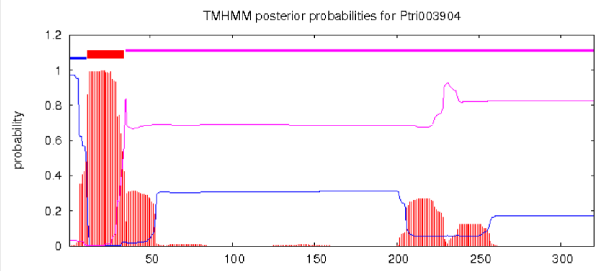

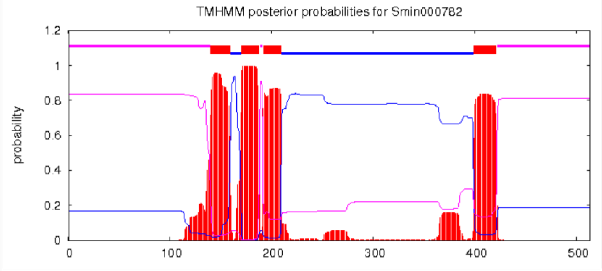

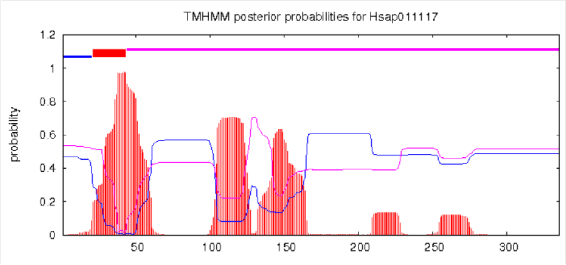

DGAT2 Clade II

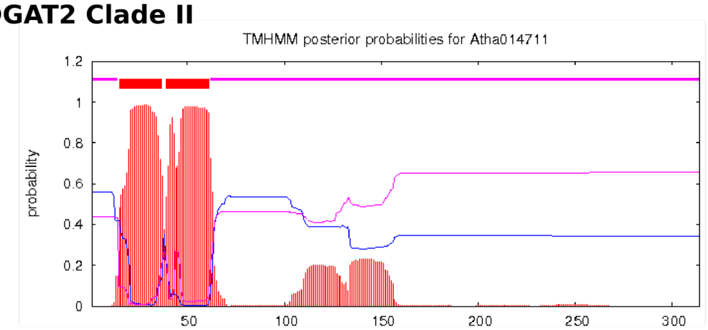

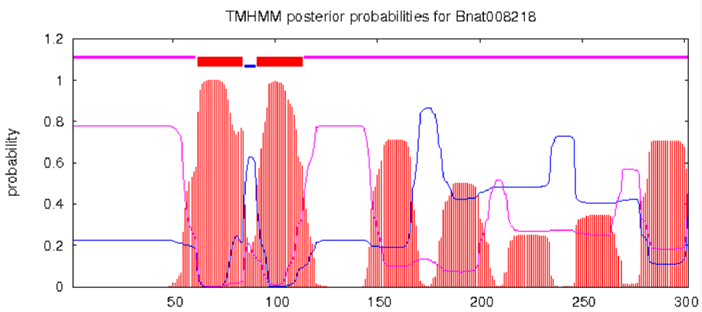

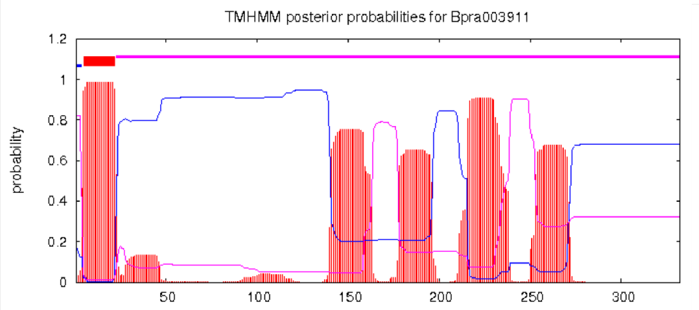

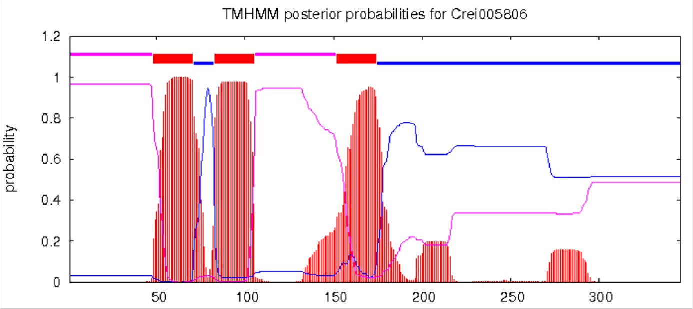

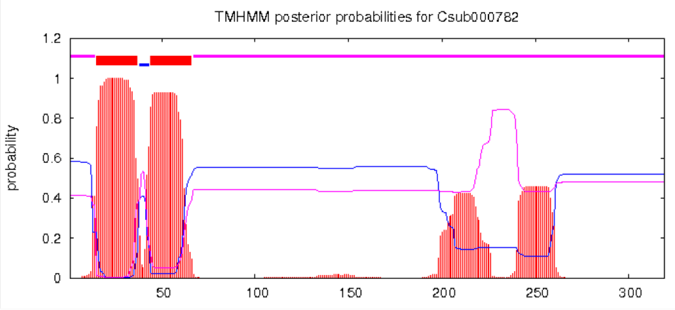

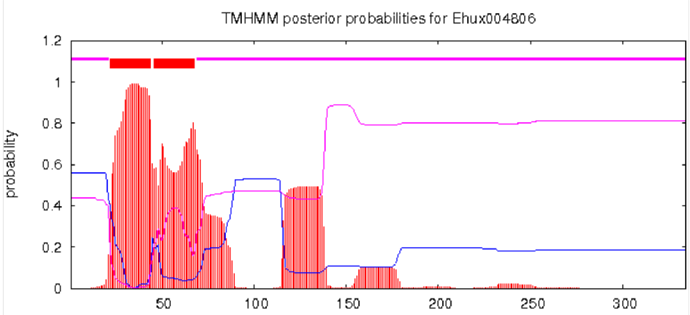

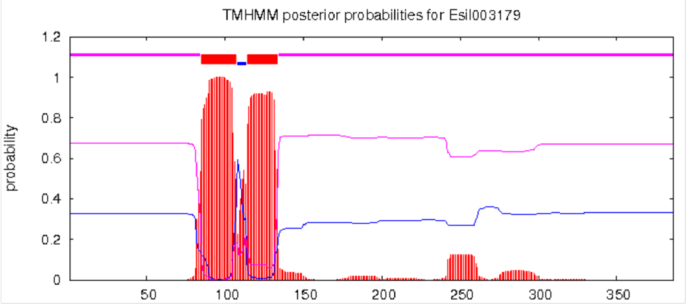

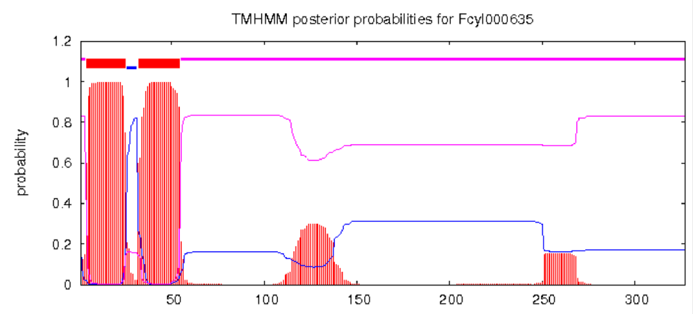

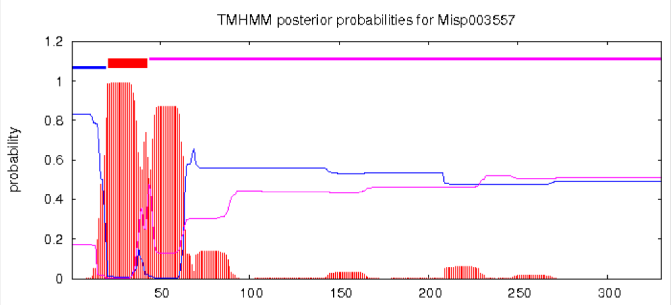

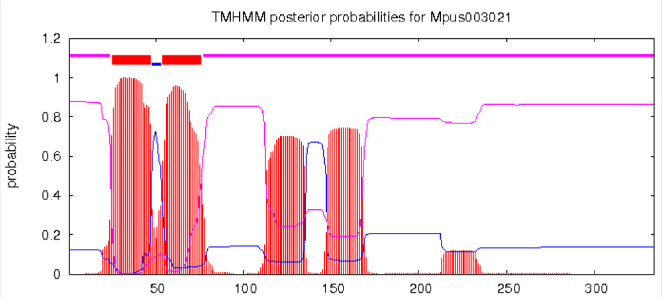

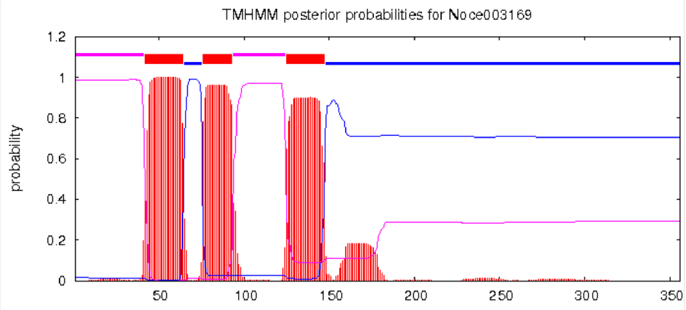

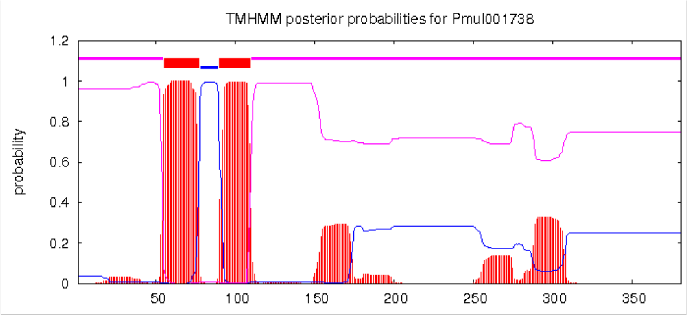

DGAT2 Clade III

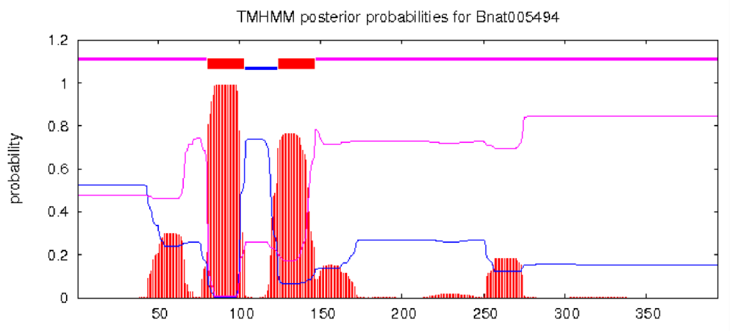

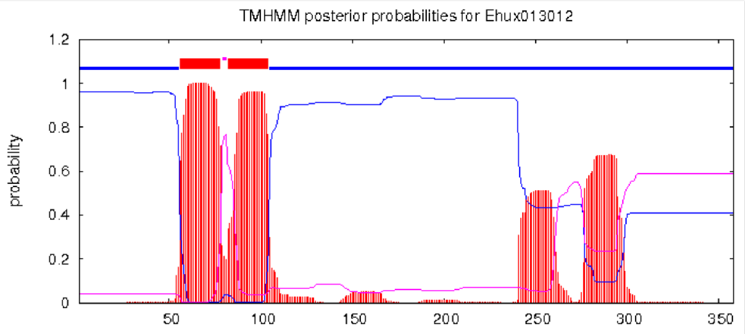

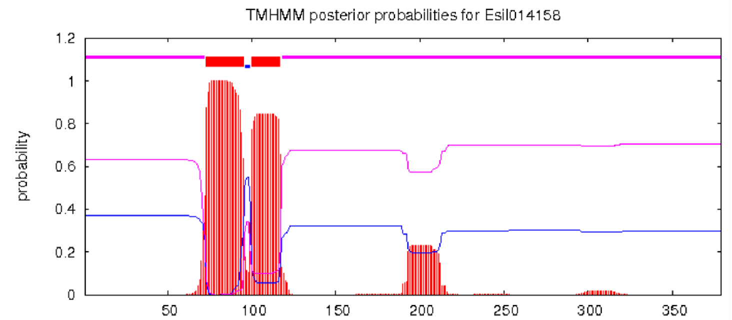

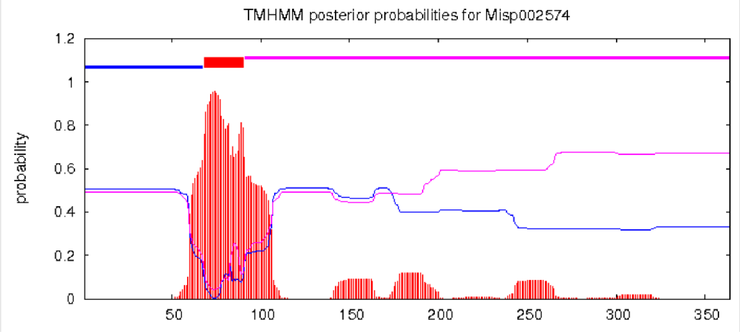

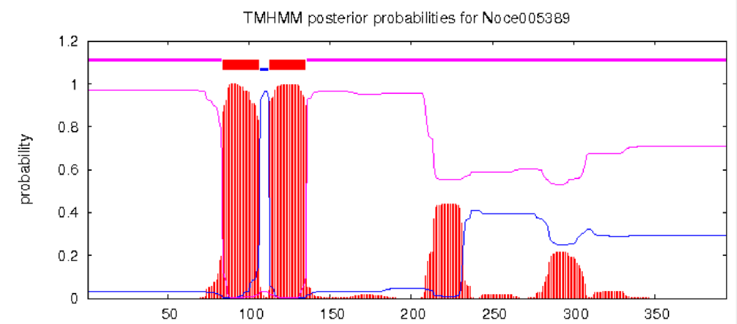

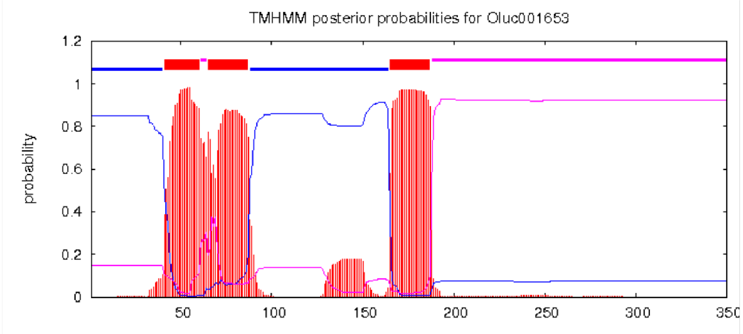

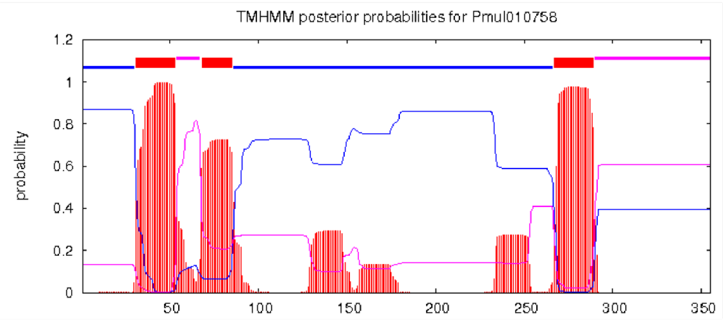

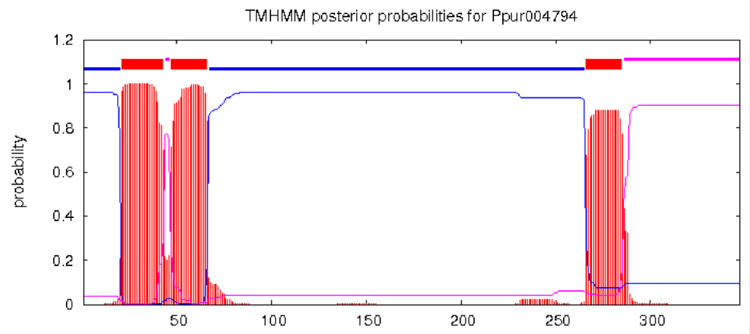

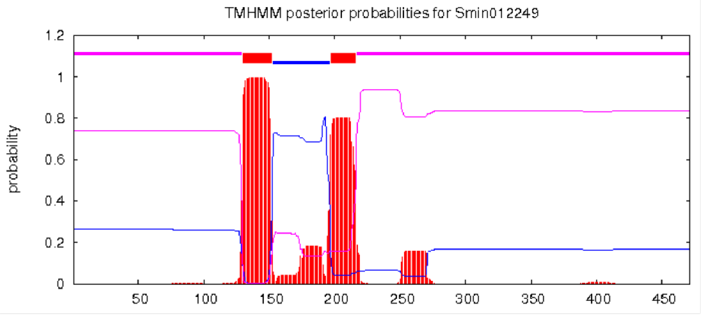

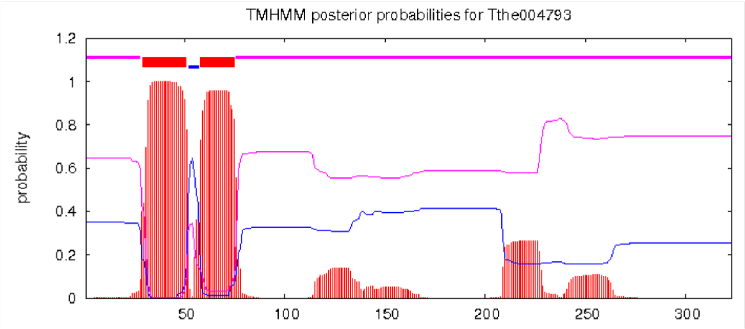

DGAT2 Clade IV

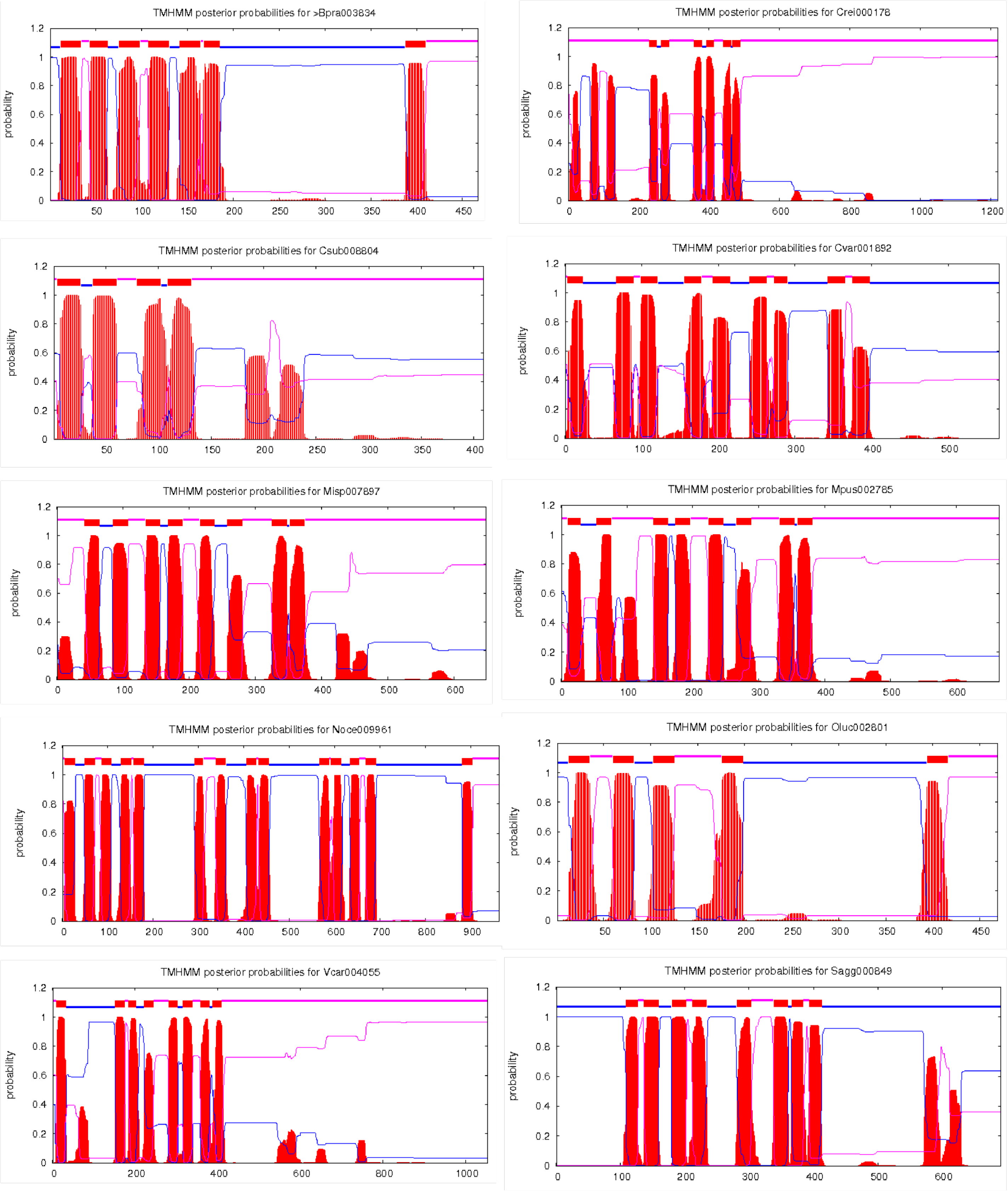

Additional File 4. Transmembrane segment prediction of algal DGAT2 sequences.
Selected algal DGAT2 sequences from the four clades (I-IV) were used for the prediction of transmembrane segments with TM-HMM. The sequences from each clade that were either significantly shorter or longer than conventional DGAT2 proteins, as well as those that did not have a starting methionine, were eliminated prior to the analysis. *Homo sapiens* (Hsap, Clade I), *A. thaliana* (Atha, Clade II), *Tetrahymena thermophila* (Tthe, Clade III) and *Schizochytrium aggregatum* (Sagg, Clade IV) sequences are shown as reference of DGAT2 from other organisms. TM-HMM plots are shown, which include number, amino acid position and posterior probabilities of predicted transmembrane segments (red) and location of the connecting loops (blue for inside loops and pink for outside loops).
