# Supplementary material for: Analysis of triglyceride synthesis unveils a green algal soluble diacylglycerol acyltransferase and provides clues to potential enzymatic components of the chloroplast pathway
Source: BMC Genomics. 2017 Mar 9;18:223. doi: 10.1186/s12864-017-3602-0 (PMC5343412; doi:10.1186/s12864-017-3602-0)
Supplement: Additional file 5: — Hydrophobicity and transmembrane segment analysis of C. reinhardtii DGATs. (PDF 418 kb) [file 12864_2017_3602_MOESM5_ESM.pdf]

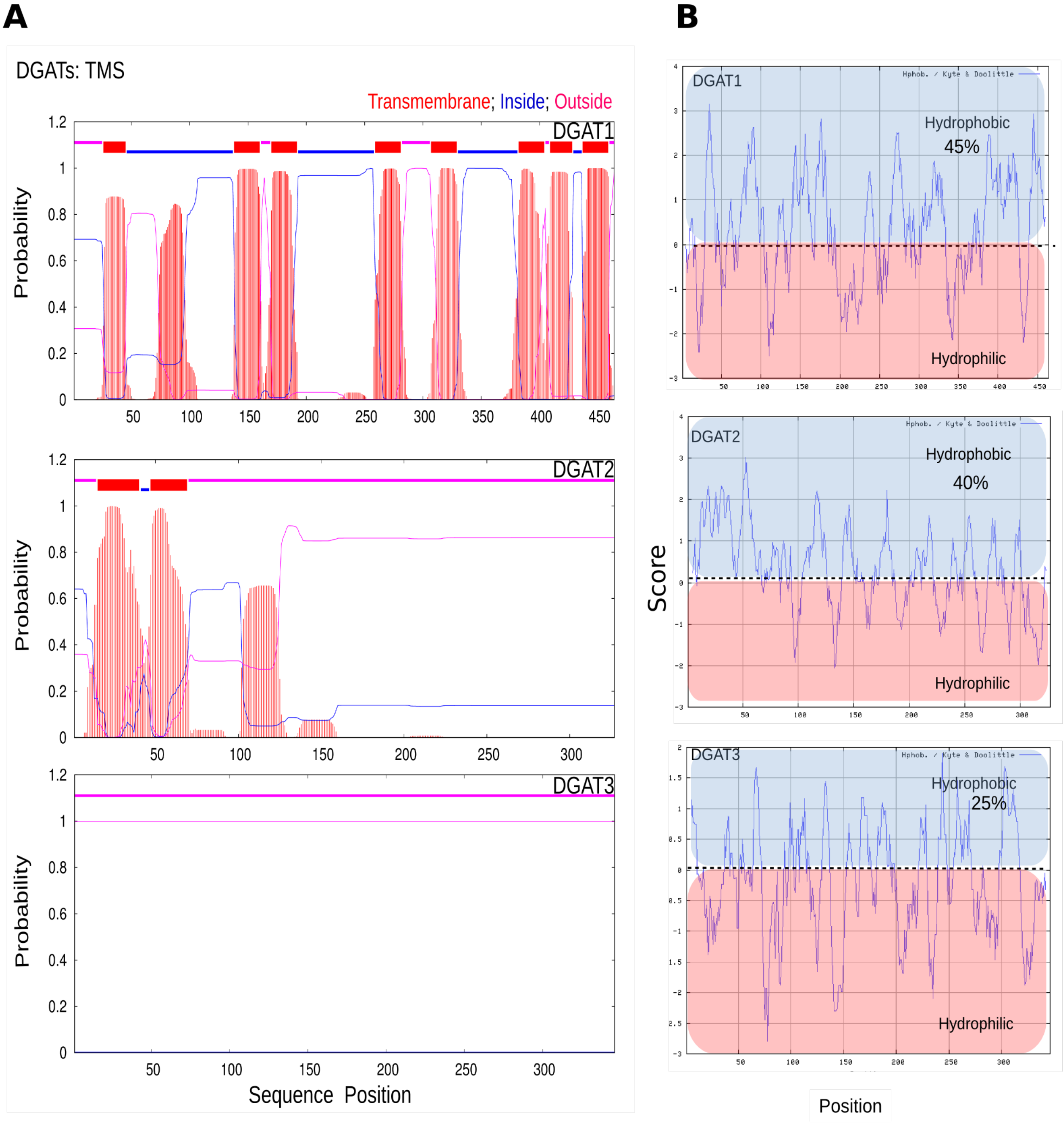

Additional File 5. Hydrophobicity and transmembrane segment analysis of *C. reinhardtii* DGATs.
A. Graphics showing TM-HMM transmembrane segment (TMS) prediction analisis of *C. reinhardtii* DGAT1, DGAT2 (DGTT4) and DGAT3 protein sequences. The presence and location of TMSs is indicated in red, the blue and pink segments and plots show the predicted amino acid residue distribution across the membrane. The *y* axes correspond to the posterior probabilities associated with the analysis. *B.* Hydropathic plots from Kyte and Doolittle probability analysis. Plots show hydrophobic domain distribution along protein sequences for *C. reinhardtii* DGAT1, DGAT2 and DGAT3. Blue indicates hydrophobic regions, pink indicates hydrophilic regions. The percentages of hydrophobic regions of each protein are shown.
